# Pullulanase: unleashing the power of enzyme with a promising future in the food industry

**DOI:** 10.3389/fbioe.2023.1139611

**Published:** 2023-06-28

**Authors:** Bindu Naik, Vijay Kumar, S. K. Goyal, Abhishek Dutt Tripathi, Sadhna Mishra, Per Erik Joakim Saris, Akhilesh Kumar, Sheikh Rizwanuddin, Vivek Kumar, Sarvesh Rustagi

**Affiliations:** ^1^ Department of Food Science and Technology, Graphic Era (Deemed to be University), Uttarakhand, India; ^2^ Himalayan School of Biosciences, Swami Rama Himalayan University, Dehradun, India; ^3^ Department of Agricultural Engineering, Institute of Agricultural Sciences, Banaras Hindu University, Varanasi, India; ^4^ Department of Dairy Science and Food Technology, Institute of Agricultural Sciences, Banaras Hindu University, Varanasi, India; ^5^ Faculty of Agricultural Sciences, GLA University, Mathura, India; ^6^ Department of Microbiology, Faculty of Agriculture and Forestry, University of Helsinki, Helsinki, Finland; ^7^ Department of Food Technology, UCLAS, Uttaranchal University, Dehradun, India

**Keywords:** pullulanase, solid-state fermentation, agro-waste, industrial applications, fungi

## Abstract

Pullulanases are the most important industrial group of enzymes in family 13 glycosyl hydrolases. They hydrolyze either α-1,6 and α-1,4 or both glycosidic bonds in pullulan as well as other carbohydrates to produce glucose, maltose, and maltotriose syrups, which have important uses in food and other related sectors. However, very less reports are available on pullulanase production from native strains because of low yield issues. In line with the increasing demands for pullulanase, it has become important to search for novel pullulanase-producing microorganisms with high yields. Moreover, high production costs and low yield are major limitations in the industrial production of pullulanase enzymes. The production cost of pullulanase by using the solid-state fermentation (SSF) process can be minimized by selecting agro-industrial waste. This review summarizes the types, sources, production strategies, and potential applications of pullulanase in different food and other related industries. Researchers should focus on fungal strains producing pullulanase for better yield and low production costs by using agro-waste. It will prove a better enzyme in different food processing industries and will surely reduce the cost of products.

## 1 Introduction

Starch processing enzymes are one of the well-known classes of microbial enzymes used in operations like brewing, baking, medicines, etc. The family of enzymes known as amylases is mostly employed to hydrolyze starch (Rubilar et al., 2008; Liu and Kokare, 2023). The four different groups of starch-processing enzymes include exoamylases, endoamylases, transferases, and debranching enzymes. Pullulanase and α-Amylase are the most efficient enzymes in this category (starch hydrolyzing enzymes) accounting for around 30% of the global enzyme market (Paul et al., 2021). During the saccharification process, they hydrolyze the starch’s glycosidic linkages to glucose, maltose, and maltotriose.

The food industry and other sectors have found substantial uses for these products. As pullulanase hydrolyzes pullulan, starch, and other carbohydrates by breaking both the 1,6 and 1,4 glycosidic bonds (Hii et al., 2012), it is more crucial for industrial application than the other hydrolases due to its special characteristic. There are five different types of pullulanase, including pullulanase types I and II and pullulan hydrolase types I, II, and III (Table 1) (Kahar et al., 2022). Various Pullulanases their substrate and products are given in Table 1. For the first time, the pullulanase enzyme was discovered in *Klebsiella* (formerly known as *Aerobacter aerogenes*) (Wallenfels et al., 1966). To produce maltotriose (from pullulan) and linear oligosaccharides (from branching oligosaccharides), pullulanase type I is used which hydrolyzes α-1,6 glycosidic bonds (Chen et al., 2022). Nevertheless, this enzyme did not hydrolyze glucans, amylose, glycogen, or panose (Kashiwabara et al., 1999). Maltotriose is produced by Type II Pullulanase (Amylopullulanases) hydrolyzing α-(1,6) links in pullulan, while glucose and maltose are produced by hydrolyzing α-(1,4) linkages in starch and maltotriose, respectively (Roodi et al., 2017; Kahar et al., 2022). Pullulan’s α-(1,4) linkages are hydrolyzed by Type I Pullulan Hydrolases (Neopullulanases), which results in panose (Wang et al., 2022). *Aspergillus niger* has been found to produce pullulan hydrolase type II (also known as isopullulanases), which forms isopanose by hydrolyzing α-(1, 4)-D glycosidic linkage (Aoki and Sakano, 1997). Pullulan Hydrolases Type III is the final group and hydrolyzes both the α-1,6 and α-1,4 glycosidic linkages in pullulan to produce the main sugars maltotriose, panose, and maltose. To produce maltotriose and maltose, it also breaks down starch, amylopectin, and amylose (Toor et al., 2020; Kahar et al., 2022). Due to the existence of catalytic sites, the pullulan hydrolase type III enzyme’s primary three acidic residues—two aspartic and one glutamic acid—are primarily involved in its catalytic activity. *Thermocococcus* aggregates have been found to contain these novel pullulan hydrolase types (Niehaus et al., 2000; Hii et al., 2012). The enzymes that consecutively cleave α-1, 4 glucoside linkages from the polymer β-glucans like pullulans to create glucose are exoglucanases (EC. 3.2.1.3) and glucoamylases (CGA) (Sharma et al., 2022). Due to its ability to hydrolyze α-(1,4) and α-(1,6) links in pullulan and starch, pullulan hydrolase type III is of greater interest and significance. Nevertheless, combinations of enzymes must be used for the same process. Pullulan hydrolase type III is thus used to increase the industrial process’ economic viability. However, Due to low yield concerns, there are very few reports on native strains producing pullulanase. Finding innovative, highly productive pullulanase-producing microbes has become crucial due to the rise in pullulanase demand. Moreover, the industrial production of pullulanase enzyme is severely constrained by its high manufacturing cost and low yield. By choosing agro-industrial waste, the production cost of pullulanase (in solid-state fermentation, SSF) can be reduced. Many agro-industrial wastes, including sawdust, maize cobs, rice bran, green gram husk, wheat straw, soy hull, grape wine trimmings dust, sweet sorghum pulp, sugarcane bagasse, banana peel, palm oil mill waste, etc., have been used to produce numerous primary and secondary metabolites in SSF (Ravindran et al., 2018; Naik et al., 2019; Kumar et al., 2022). The utilization of agricultural wastes helps to address environmental issues and lower the cost of enzyme production. Moreover, there are not many reports on pullulanase from fungi.

**TABLE 1:** Various Pullulanases their substrate and products.

Sl. No.	Accepted name	EC number	Other names	Systematic name	Bond hydrolyzed	Substrates	Products	References
1	P-I	3.2.1.41	*α*-dextrin 6 glucanohydrolase, pullulan α-1,6-glucanohydrolase, pullulanase, α-dextrin endo-1,6-α-glucosidase	Pullulan 6-α-glucanohydrolase	α-(1,6)- D-glucosidic linkages	pullulan, amylopectin, glycogen, α-and β-limit dextrinsother branched oligosaccharide	α-and β-limit dextrins, Maltose, maltotriose, and linear oligosaccharides	[Lee and Whelan, 1966; Bender and Wallenfels, 1966; Man
			R-enzyme; pullulan 6-glucanohydrolase					Bertoldo and Antranikian, 2002; Guo et al., 2018
2	P- II	3.2.1.41	Amylopullulanase		α-(1,6) and α-(1,4) D-glucosidic linkages	Pullulan, Poly and oligo saccharides, starch, amylopectin	Maltotriose, glucose, maltose	Duffner et al., 2000; Roy and Gupta, 2004; Lévêque et al., 2000
3	PH-1	3.2.1.135	Neopullulanase	pullulan 4-D-glucanohydrolase	α-(1,4) D-glucosidic linkages	Pullulan	Panose	Ara et al., 1995; Imanaka and Kuriki, 1989; Sunna et al., 1997
4	PH-II	3.2.1.57	Isopullulanase	pullulan 4-glucanohydrolase (isopanose-forming)	α-(1,4) D-glucosidic linkages	Pullulan, Panose	Isopanose, isomaltose and glucose	
5	PH-III		-		α-(1,6) and α-(1,4) D-glucosidic linkages	Pullulan, Starch, amylose and amylopectin	Panose, maltose and maltotriose	Niehaus et al. (2000)

Symbols used: P-I, Pullulanase type I, P-II, Pullulanase type II, PH-I, Pullulan hydrolase type I, PH-II, Pullulan hydrolase type II, PH-II, Pullulan hydrolase type III.

## 2 Sources of pullulanase

Due to the high demand for microbial enzymes in various industrial processes, the quest for new enzyme-producing microorganisms as the primary sources of new biocatalysts has been increased in recent times. The research is driven mainly by the vast diversity of microorganisms, both phylogenetics, and ecogeographic (Soares et al., 2012). The enzymes that degrade pullulan (Pullulanase) has been reported from plants [Solarium tuberosum L. (Potato; Ishizaki et al., 1983), Spinacia oleracea L. (Spinach; Renz et al., 1998), *Hordeum vulgare* (Barley; Møller et al., 2015), and *Manihot esculenta* Crantz (Cassava; Wangpaiboon et al., 2023)], yeasts [Clavispora lusitaniae ABS7 (Dakhmouche Djekrif et al., 2021)], fungi [Aureobasidium pullulans (Hamidi, et al., 2019)]and bacteria [mesophilic (*Bacillus macerans*, and *Bacillus acidopullulyticus*), thermophilic and hyperthermophilic bacteria (*Clostridium thermosulfurogenes*, *Bacillus stearothermophilus,* and *B. naganoensis*) (Gomes et al., 2003; Gangadharan and Sivaramakrishnan, 2009; Song et al., 2017)]. In the saccharification and brewing process, the pullulanase used has been obtained from *Bacillus* spp and *Klebsiella* spp. (Prabhu et al., 2018). Most of the pullulanase reported are from bacterial origin while very little from fungi. Hence further research on fungi producing Pullulanase is needed. Several pullulanase-producing microorganisms are mentioned in Table 2.

**TABLE 2:** Source microorganisms and properties of pullulanase.

Types of pullulanase	Organisms	Enzyme properties			References
		Optimum T (˚C)	OptimumpH	Molecular weight (kDa)	
	*Bacillus stearothermophilus*	60–65	6.0	62	Kuriki et al. (1988)
Pullulanase Type I	*Fervidobacteriumpennavorans*Ven5	65–85	6.0	83, 93	Bertoldo et al. (1999)
	*Paenibacilluspolymyxa*	35	6.0	96	Wei et al. (2015)
	*Thermusculdophilus* GK-24 b	75	5.5	65	Kim et al. (1999)
Pullulanase type II	*Thermoanaerobacterethanolicus*	90	5.5	-	Mathupala et al. (1993)
	*Pyrococcusfuriosus*	50	6.0	90	Zona et al. (2004)
	*Bacillus cereus*	55	6.0	NA	Ling et al. (2009)
	*Pyrococcuswoesei*	100	6.0	90	Rüdige et al. (1995)
	*Thermotoga maritima*	90	7.5	58	Domań-Pytka and Bardowski (2004)
	*Streptococcus infantarius*	30–45	6.8–8.0	246.3	Rodrigues et al. (2013)
	*Lactobacillus cripatus*	37	3.5–4.0	-	Woolston et al. (2021)
Pullulan hydrolase type I	*Alicyclobacillus acidocaldarius*	55	5.5	66	Domań-Pytka and Bardowski (2004)
	*Bacillus stearothermophilus*	60–70	-	69	Domań-Pytka and Bardowski (2004)
	*Thermoactinomyces vulgaris*	40	5–6	65	Domań-Pytka and Bardowski (2004)
Pullulan hydrolase type II	Aspergillus niger	30–40	3.4–4.0	-	Sakano et al. (1971)
Pullulan Hydrolase type III	*Thermococcus aggregans*	100	6.5	83	Niehaus et al. (2000)
	*Thermococcus kodakarensis*	95–100	3.5–4.2	84	Ahamad et al., 2013; Toor et al., 2020

Symbols used: NA, data not available; T-temperature.

### 2.1 Structural characteristics of pullulanase

Pullulan is described as a polymer of (1 → 6) linked maltotriose subunits since it converted a yeast (*A. pullulans*) α-glucan containing α-(1 → 6) bonds into maltotriose. Sometimes, the partial acid hydrolysis of Pullulan generates isopanose, and panose. Therefore, often suggested as a polymer of isopanose or panose (Singh et al., 2008). The metabolic pathways of pullulan generation as well as the fundamental structure of the biopolymer are influenced by the wide range of environmental factors, and the different traits found in various strains (Shingel, 2004). Pullulan’s particular physical characteristics, pressure mouldings, adhesive qualities, oxygen-impermeable films and ability to form fibres, are all corresponding to its peculiar linking pattern. Chemical derivatization can be used to regulate or add reactive groups aiding to pullulan’s solubility (Singh et al., 2008).

The structural framework of pullulanases includes a carbohydrate-binding module (CBM- CBM41, CBM48, and CBM68), a C-terminal domain and a catalytic domain. According to Janeek et al. (2017), the CBM domain at the N-terminal, is crucial for the binding of enzymes to polysaccharide substrates and for facilitating the hydrolysis process. In order to keep the enzyme in their active shape, certain CBMs also serve as a catalytic unit (Armenta et al., 2017). Additionally, several unidentified domains (X domains, such as X25 and X45) are found at the N-terminal of pullulanases that may attach to the d-glucan substrate connected via 1,4 and 1,6 glucosidic linkages, supporting their binding functions as CBMs (Turkenburg et al., 2009). The conserved N-terminal CBMs and unconserved C-termini with unknown functions allows such areas as attractive for engineering to modulate the catalyses of pullulanases (Xu et al., 2021).

According to Nisha and Satyanarayana (2016), the catalytic domain can be stabilised via interactions between the hydrophobic area of the N-terminal domain and the usual beta-sheet structure of the C-terminal domain of GH13. Pullulanase obtained from *Anoxybacillus* sp., LM18-11 (PulA) possess two oligosaccharide molecules in the catalytic domain when arranged in parallel binding mode. Moreover, two more oligosaccharide molecules were discovered in the catalytic domain’s loop between the third beta-strand and third alpha-helix and the carbohydrate-binding motif. This structural arrangement has been reported to provide thermostability (Xu et al., 2014).

**FIGURE 1:**
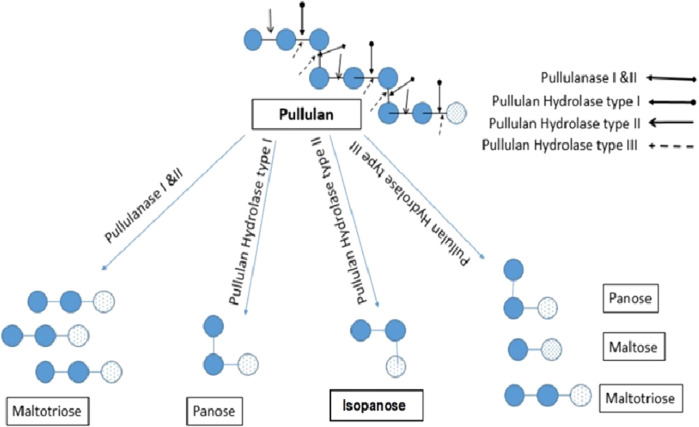
Catalytic pattern of different Pullulanase. Pul I and II hydrolyzes α (1,6) glycosidic bond to produce maltotriose; Pullulan hydrolase I hydrolyzes α-(1,4) glycosidic bond yielding panose; pullulan hydrolase II catalyzes α- (1, 4)-D glycosidic bond to produce isopanose; pullulan hydrolase III hydrolyzes *α*-1,4- as well and *α*-1,6-glucosidic bonds to produce panose, maltotriose, and maltose.

Almost all pullulan degrading enzymes consist of highly preserved I-IV fields that are active centers and prevent substrate sites for amylases (Liebl et al., 1997). Additionally, Asp and Glu are found in the catalytic site which plays a significant role in catalytic activity (Xu et al., 2021). Asp-206, His-210, and His-296 are the substrate-binding sites that display a significant role in the catalysis of enzymes (Kuriki et al., 1988). The cleavage site by the different groups of pullulanase is demonstrated in Figure 1. Several techniques such as Infra-red spectroscopy, proton and carbon-13 NMR spectroscopy, Fourier transform infrared spectroscopy and Raman spectroscopy are employed to study the structure of various pullulanase.

Pullulanases are recently studied for their debranching character along with the high temperature–pressure, autoclaving-cooling cycles, and storage temperature on resistant formation in cassava, potato, corn and rice starches (Babu and Satyanrayana, 1995; Miao et al., 2009; Bodjrenou et al., 2022) and are frequently employed in the industrial starch conversion process to saccharify starch, which lowers the need for glucoamylase and shortens the overall reaction time (Nisha and Satyanarayana, 2016).

### 2.2 Substrates of pullulanase

Pullulan (α-1, 4-glucan or α -1,6-glucan): *Aureobasidium pullulans* produce a polysaccharide known as Pullulan (Kim et al., 2000)**.** The structure of pullulan is given in Supplementary Figure S1A. The maltotriose units are joined together by α-1, 6-glycosidic bonds (Pandey et al., 2021) while isopanose is linked by 1, 4-glycosidic bonds to form Pullulan (Leathers, 2003). The α-1, 4- to α-1, 6-glycosidic bond ratio are 2:1. It has broad applications in pharmaceutical industries and the food-processing industries and is also used to study substrate specificity of amylase (Singh et al., 2008; Taniguchi and Honnda, 2009).

#### 2.2.1 Starch

Starch is a major storage biomolecule found in plants. It is one of the important raw materials for various industries like sugar syrup, confectionary, fuel industry, detergent industries, etc (Jòzef, 2007). Starch is the second most abundant heterogeneous plant-produced polysaccharide after cellulose. It is a water-insoluble polysaccharide that contains two polymers, amylose (linear polymer of glucose, Supplementary Figure S1B) and amylopectin (branched structure, Supplementary Figure S1C) (Swinkels, 1985), exhibiting different solubility in water. Amylopectin gives a crystalline structure due to regular branching and in this, both α-1, 4- and 1, 6 glycosidic bonds are found. In amylose, glucose molecules are linearly attached by α-1, 4 glycosidic bonds (Park et al., 2018). Starch hydrolysis by amylases results in dextrans and a reduced yield of glucose. This limitation can be overcome using pullulanase (Poliakoff and Licence, 2007).

#### 2.2.2 Glycogen

It is a water-soluble complex polysaccharide mainly reported from both animals and microorganisms. It is a more complex polysaccharide than starch and pullulan. It also contains both α-1, 4- and 1, 6 glycosidic bonds like amylopectin (Supplementary Figure S1D).

## 3 Approaches used to increase pullulanase yield

### 3.1 Solid states fermentation (SSF) *versus* submerged fermentation (SmF)

**FIGURE 2:**
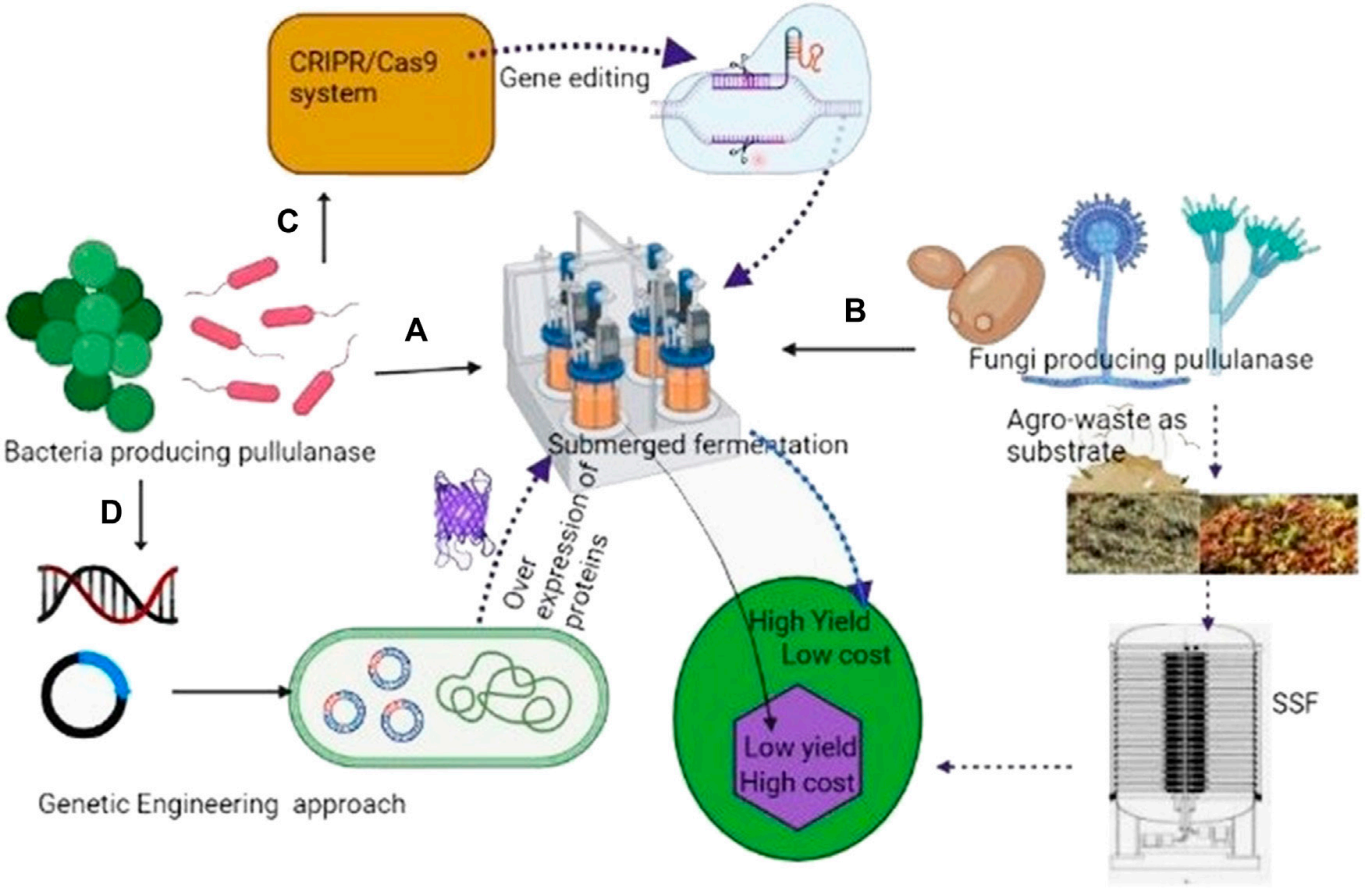
Approaches used to increase yield of pullulanase. **(A)**- Pullunanse production by bacteria in Smf (costly); **(B)**-Fungi producing pullulanase using agro-waste as a substrate (cost effective); **(C)**-high production of pullulanase by CRISPR engineering.; **(D)**-Heterologous expression of pullulanase genes for high yield (genetic engineering).

In fermentation, process microbes convert solid or liquid substrates into a variety of products. Based on the physical state of the substrates, the major fermentation processes used for the industrial production of enzymes include SmF and SSF (Figure 2). SmF is carried out in a nutrient media with soluble or insoluble substrates in excess of water via batch, fed-batch or continuous operational modes. Higher working volume in SmF is mainly achieved by water, thus creating homogenous conditions and facilitating the proper mixing, modeling, and design of bioreactors as well completes control of the process for better performance (López-Gómez and Venus, 2021). Heat mass transfer constraints are not an issue with SmF operations, which can be easily scaled up and automated (Sharma et al., 2016). The low productivity, high production cost, and complexity of the medium are, nevertheless, the key downsides of SmF methods (Babbar and Oberoi, 2014).

On the other side, SSF is carried out in the presence of low moisture which supports the growth of molds, and water activity of less than nine does not support the growth of bacteria (Gowthaman et al., 2001). A very small particle of substrate minimizes the availability of void space, reduces aeration, and affects the downstream processing of the products (Pandey et al., 2000).

The pH of the substrate is a critical factor in fermentation. In SSF, the substrate pH is maintained initially or the buffering action of the substrate mainly the protein-rich substrate avoids the major changes in pH of the substrate during fermentation (Lonsane et al., 1992). Aeration is important in proper heat transfer of the medium for maintaining the temperature and moisture of the medium. Proper aeration removes CO_2_ resulted due to respiration and product formation. The removal of CO2 and improved oxygen availability increase the yield. However, the excess agitation may damage the hypha structure of the fungi (Lonsane et al., 1992). Proper agitation avoids the attachment of the microbe to the substrate and gives an improved yield.

During SSF heat is generated due to respiration and the metabolic process of microbes. The removal of heat by aeration and frequent spraying of sterile cold water reduces the temperature of the medium (Krishna, 2005). For better productivity, it is important to maintain moisture and aeration. SSF has various advantages over SmF, including reduced catabolite repression and substrate inhibition, superior enzyme harvests and volumetric outputs, low energy consumption, prolonged product stability, no discharge of organic wastewater, and low production costs (Kumar et al., 2020; Salgado-Bautista et al., 2020). SSF has gained more importance due to the use of low-cost Agro-waste substrates (Figure 2) (Kumar et al., 2020).

Molds have been significantly exploited for their extracellular enzymes in SSF as compared to other groups of microbes because of their ability to grow in solid substrates (Archana and Satyanarayana, 1997). SSF has several advantages such as a cost-effective medium (simple composition), fewer effluents released, releases a negligible quantity of effluent, and pollution problems are reduced. Moreover, concentrated products are obtained in SSF as compared to SmF (Gajdhane et al., 2016; Lopez-Gomez and Venus, 2021).

### 3.2 Agro-waste-based substrates used for the production of the enzyme in SSF

The agro-wastes are a rich source of carbon that can be used to produce both microbial biomass and metabolites. It can act as cheaper fermentation media for lowering the costs of enzyme production (Martins et al., 2011). Industrial biotechnology advances give potential possibilities to use agro-industrial residues economically. Rice bran and wheat bran are important by-products of the rice and wheat processing industries. These two by-products can be successfully utilized to produce various value-added products (Ahmad et al., 2013). *Phaseolus vulgaris* (Local red kidney beans), *Pistia stratiotes* (water cabbage), *Eichhornia crassipes* (water hyacinth), and *Ipomoea batatas* (sweet potato) were recently as novel substrates to produce pullulanase (Velhal et al., 2014; Šelo et al., 2021). The various agro-wastes used to produce glucose hydrolase 13 families are given in Table 3.

**TABLE 3:** Agro-waste used for the production of GH13 family in SSF.

Substrates	Enzymes	Micro-organisms	References
Sweet potato/Water hyacinth	*Pullulanase*	*Auerobasidium pullulans*	Velhal et al. (2014)
Wheat bran	*Pullulanase*	*Clostridium thermosulfurogenes SV2*	Reddy et al. (1999)
Wheat bran	Pullulanase	*Clostridium thermosulfurogenes* SVM17	Mrudula et al. (2011)
Rice bran + corn bran	Pullulanase	K. aerogenes NCIM2239	Prabhu et al. (2018)
Wheat bran	Amylase	*Bacillus coagulans*	Babu and Satynarayana (1995)
Wheat bran	Amylase	Thermomyceslanuginosus	Kunamneni et al. (2005)
Wheat bran	Glucoamylase	*Aspergillus* sp	Fadel et al. (2020)
Sugarbeet pulp	Glucoamylase, α-amylase	*Aspergillus oryzae*	Fadel et al. (2020)
Sugarcane pulp	Amylase	*Aspergillus* and *Trichoderma* spp.	Desgranges and Durand (1990)
Rice bran	α-amylase	*Rhizopus oryzae*	Kaur et al. (2015)
Groundnut oil cake	Amylase	*Aspergillus niger*	Suganthi et al. (2011)
Pomegranate peel	Amylase	*Aspergillus fumigatus*NTCC1222	Singh et al. (2013)
Millet, oat, tapioca, and arum	Isoamylase	*Rhizopus oryzae*PR7	Ghosh and Ray (2011)
Garden pea peel	Glucoamylase	*Aspergillus niger*	Banerjee and Ghosh (2017)
Wheat bran	Pullulanase	*Aspergillus flavus*	Naik et al., 2019; Naik et al., 2021

The production of pullulanase in SSF by bacterial systems is also being researched. SSF is chosen over Smf for the synthesis of pullulanase because of its many benefits. SSF uses straightforward and affordable media, making it an efficient and acceptable solution. Moreover, SSF has fewer chances of contamination because most contaminants cannot survive in the absence of low water activity. After process optimization in SSF, bacterial species such as *Bacillus licheniformis* (Khalaf and Aldeen., 2013) and *Clostridium thermosulforegenes* (Mrudula et al., 2011) produced pullulanase effectively. The feasibility of using SSF to produce the enzyme on a large scale, however, was not investigated (Akassou and Groleau, 2019). From the above table, it was evident that the enzymes such as amylases and glucoamylases have been produced from fungi in SSF by utilizing different agro-based waste, but little information is available on pullulanase production from fungi in SSF. Hence, the need of the hour is to find fungi producing pullulanase.

### 3.3 Genetic engineering

In industry, pullulanase is widely used, yet the yield of this enzyme produced by bacteria is small. To enhance the amount of pullulanase production by bacteria, researchers used transcriptome data to locate strong promoters (Figure 2). In general, systematic biology can be used to create metabolic models, predict gene function and protein structure, and direct metabolic engineering (Lam et al., 2012; Juhas et al., 2014). Genetically engineered bacterial systems have a higher yield than wild strains.

For a higher yield of proteins, a strong promoter is required because it is well-known that protein yield and promoter strength are closely related (Blazeck et al., 2012). The cytidine deaminase (ccd) promoter P43 of *B. subtilis* is the most well-known promoter and has been utilized to express *GFP* (Kong et al., 2009), -galactosidase, staphylokinase (Kim et al., 2008), and alkaline protease (Kim et al., 2008). Using a promoter trapping technique, Yang et al. identified a potent *B. subtilis* promoter (Plaps) that is 13 times stronger than the P43 promoter. Promoters can be joined together to create multiple-promoter complexes, which have been found to increase enzyme production by up to 1.6 times and 12 times (Kang et al., 2010). Zhang et al. (2017) reported a *PhpaII-PamyQ*, dual-promoter expression system, which increased the yield from 18.7 times to 571.2 U/mL in a 3 L fermenter.

Meng et al. (2018) analyzed dual- (PsodA + fusA) and triple- (PsodA + fusA + amyE) promoter-constructed strains in a shake flask and found the highest yield of 163 U/mL and 336 U/mL respectively which was 2.29 and 4.73 times higher than that of the strain having PamyE. The strain including PsodA + fusA + amyE also displayed a maximum activity of 1,555 U/mL, 21.9 times more than that of the PamyE strain produced in flasks in a 50L fermenter.

Several researchers have constructed recombinant strains to improve the yield of pullulanase. Coleman. (1993) constructed a recombinant strain of *E. coli* for the expression of *Thermoanaerobium brockii* amylopullulanase gene and recorded a higher yield (0.80–1.0U/mL) than the wild strain (0.23U/mL). Kim et al. (2008) reported a higher yield from the recombinant *Lactobacillus plantarum* (3.5U/mL) strain. Similarly, the pullulanase gene from *Paenibacillus*, *Geobacillus*, *Bacillus subtilis*, *Bacillus naganoensis,* and *Bacillus acidopullulyticus* has been expressed in recombinants for higher yields (6.48 U/mL, 17.35 U/mL, 269.1 U/mL, 684 U/mL respectively). The greatest pullulanase activity produced by recombinant *Bacillus subtilis* strain WS9PUL was much higher than that of wild strains and recombinants of *E. coli* (1567.9 U/mL), *B. choshinensis* (1005.8 U/mL), or *P. pastoris* (350.8 U/mL) (Zou et al., 2014; Zou et al., 2016a; Zou et al., 2016b). Pullulanase activity of 5951.8 U/mL was attained by recombinant strain WS9PUL, which is the highest activity ever recorded (Zhang et al., 2020a). Although, other bacterial species have also been engineered to produce pullulanase *Bacillus subtilis* system is always a choice for heterologous production of proteins because it lacks an outer membrane and absence of significant bias codon usage which is important for secretion, transcription, and translation process (Beaulieu et al., 2005; Su et al., 2010).

In this context, various authors reported the enhanced production of pullulanase from recombinant *Bacillus subtilis* (Zhang et al., 2020b; Pang et al., 2020). Efficient enzyme and metabolite-producing strains produced by CRISPR engineering exist today, highlighting the technology’s enormous potential. Recently by using CRISPR technology, Zhang et al. (2021) replaced the native signal peptide of pullulanase with that encoded by ywtF which increased the yield of pullulanase by 12%. The highest extracellular pullulanase production was 8037.91 UmL^-1^. This study emphasizes the value of signal peptide optimization and dltB deletion in boosting extracellular protein synthesis. But these strains used high-cost ingredients in the production; hence the cost of this enzyme is high. However, if agro-waste-based substrates with high-yielding strains are used this problem can be solved. This is only possible by using fungi-producing pullulanase in SSF. The strategies used to increase the yield of pullulanase have been given in Figure 2.

## 4 Protein engineering of pullulanase

Researchers have worked extensively in the area of protein engineering in recent years to develop several key techniques including site-directed mutagenesis, physical/chemical mutagenesis, N-terminal domain truncation, C- terminal domain truncation, and N/C- terminal domain truncation in order to boost catalytic efficiency (Xu et al., 2021). It should be noted that to analyse complete structural effect of the conserved and unconserved N- and C-terminal respectively before the initiation of N/C-terminus truncation in order to enhance the catalytic performance (Xu et al., 2021). For instance, eliminating the N1 domain from *G. thermoleovorans* NP33 pullulanase increased its specific activity and thermostability while maintaining the same physiological growth conditions as wild gt-apu (Nisha and Satyanarayana, 2015). A few reports including pullulanases from *Alkalilimnicola* sp. NM-DCM-1 (Mesbah and Wiegel, 2018) and *Lactobacillus plantarum* L137 (Kim et al., 2009) showed a comparable increase in enzymatic activity after the deletion of the non-conserved C-terminus. Zhang et al. (2020b) also studied the susceptiblility to turn inactive at 50°C with a 50% reduction in its specific activity observed due to N-terminal truncation in case of pullulanase PulPB1. Another strategy of Structure/sequence-guided consensus has been proven, logically easier to create variations and mutant libraries that will promote enzyme-directed evolution and save time on experiments. The phenomenon was supported by Chang et al. (2016) and Duan et al. (2013) in *B. naganoensis* and *B. deramificans* respectively.

Various scientific reports suggested the modification of certain micro-organisms such as *Bacillus acidopiillnlyticus*, *Bacillus deramificans*, *Bacillus acidopullulyticus*, *Bacillus naganoensis*, *Bacillus cereus* FDTA 13/NTG04-B4, *Geobacillus thermoleovorans* NP33, *Alkalilimnicola* sp. NM-DCM-1, *Lactobacillus plantarum* L137, etc., revealed promising outcomes including increase in catalytic activity, substrate specificity, thermostability, tolerance to temperature, pH, and salt concentration using the mentioned techniques (Mesbah and Weigel, 2018; Chen et al., 2019; Xu et al., 2021). The procedure is quite challenging since it generates a large set of experiments and data which is difficult to process. Therefore, high-throughput screening methods, and *in silico* approaches seems reliable and can be a great alternative.

## 5 Immobilization of pullulanase

The immobilization of enzymes is an excellent method for resolving issues such as manufacturing costs and enzyme stability (Hanefeld et al., 2009; Torres-Salas et al., 2011; Tufvesson et al., 2011). The immobilized enzymes have several benefits such as it can be reused several times, being easy to separate, and highly stable. It permits improvements in the industrial environment (Mateo et al., 2007); improves reaction control, and enhances reaction rate since the concentration of enzyme is significantly higher than in common enzyme reactions with the free enzyme. On the contrary, reusing the enzyme increases the risk of contamination and growth of contaminants on simple sugars generated by pullulanase hydrolysis (Iyer and Ananthanarayan, 2008; Garcia-Galan et al., 2011; Rodrigues et al., 2013). Immobilization is achieved by encapsulation, or by cross-linking of enzymes which can be done by covalent attachment or physical adsorption to a carrier (Garcia-Galan et al., 2011; Torres-Salas et al., 2011).

### 5.1 Hydrophobic synthetic macro-porous resin

Various enzymes have been immobilized to increase enzyme stability and to improve industrial processes. Pullulanase from *Bacillus acidopullulyticus* was immobilized (covalently) into Duolite XAD761 (hydrophobic synthetic macroporous resin) by forming a Shiff base (aldimine), a link between activated carbonyl to promote free protein amino acids. It shifted the optimum pH and temperature from pH 5.0 to 5.5 to neutral and temperature between 50°C and 60°C. In comparison with the free enzyme, the immobilized biocatalyst showed increased thermal stability and improved Km values for substrate (pullulan, dextran, and soluble starch) which is approached by the steric hindrance or modifications in the native structure of the immobilized enzyme. The immobilized enzyme has been reused for 35 successive cycles (Singh et al., 2010).

### 5.2 Calcium alginate

Calcium alginate has been used to entrap various enzymes. They are water-soluble and have been used in several pharma and food industries. Using calcium alginate to entrap enzymes for the industrial process is an inexpensive, rapid, non-toxic, and versatile method (Zhang et al., 2009). Roy and Gupta (2004) entrapped Pullulanase produced from *Bacillus acidopullulyticus* in alginate beads to hydrolyze starch.

### 5.3 Magnetic chitosan beads

Pullulanase from *Klebsiella pneumoniae* was immobilized by Covalent binding using Magnetic chitosan beads as support. This approach increases its stability over a wider pH range, heat stability, and relative activity (Zhang et al., 2009).

### 5.4 PMIA membrane

By adding an immobilized enzyme (PULL@CPB) to the PMIA membrane, a novel pulluanase@chitosan porous beads/Poly (m-phthaloyl-m-phenylenediamine) (PULL@CPB/PMIA) membrane with good separation and biocatalysts properties was developed Zhang et al. (2022). After 10 continuous usages, the immobilized pullulanase activity on the membrane remained at 70.8%. As a result of the PMIA membrane’s superior ability to transport pullulanase, a variety of applications for its bioactive membrane exist in the sectors of food, medicine, and other industries.

### 5.5 Streptavidin-functionalized magnetic nanoparticles

Based on the recognition between biotin and streptavidin Long et al. (2021) developed support for pullulanase immobilization. As compared to the free enzyme, the immobilized pullulanase showed significantly better pH and heat stability while maintaining high levels of activity (85.3%). At pH 5.5, the immobilized enzyme’s relative activity (75.2%) was noticeably higher than the free enzyme’s (15.8%; p 0.01). The residual activity of the free enzyme was only 21.5% after 360 min at 60°C, but the immobilized enzyme kept more than 70.6% of its residual activity. Their findings demonstrated the significant potential for using streptavidin-coated magnetic nanoparticles as a support for the immobilization of the numerous enzymes needed for ongoing biotechnological applications.

### 5.6 Epichlorohydrin-activated agarose

Pullulanase has covalently immobilized onto epichlorohydrin-activated agarose along with trichlorotriazine and casein. The enzyme immobilized by a cross-linking technique using glutaric dialdehyde showed poor stability with a sharply decreased relative activity. Immobilization of the enzyme broadens the optimum temperature range from 30°C–45°C and pH between 3 and 7. In the case of pullulanase immobilized onto activated agarose and casein-epichlorohydrin, the maximum retained activity was obtained at pH 5. In the context of retaining the relative activity, the author concluded that agarose is a better carrier than casein (Dessouki et al., 2001).

### 5.7 Miscellaneous

Pullulanase from *Klebsiella pneumoniae* was immobilized by grafting. In this case, the shorter oligosaccharides formed from the hydrolysis of pullulan are not equivalent to that formed by the free enzymes. After 24 h and 14 days of incubation at 60^○^C, the entangled enzyme maintained 75 and 30 percent of its activity. The immobilization gives a yield of 60% with an elevated Km value (Ali et al., 2015).

## 6 Global status of enzyme

In the present day, the enzyme has broadened its era of application from feed to food and become directly or indirectly a part of everyday life via the accessories used by people to live a comfortable, healthy, and quality-based life. Microbial enzymes are popular globally for their wide industrial applications (Li et al., 2012; Choi et al., 2015). The development of the food and beverage sector and the increased need to enhance the aroma, texture, and quality of food drive the enzymes market. Increased food demand combined with favourable legislation by the government will fuel the development of this segment.

There are more than 3000 enzymes out of which 150–170 are commercially in use. The projected global market for the year 2014 was $4.2 billion. By the year 2015–2021, it may reach higher than $6.2 billion*.* In 2016 the enzyme market was USD 5 billion which will surpass 400 kilotons by 2024. By 2024 the global market will reach USD 17.50 billion (Verma, 2019). Out of the whole enzyme market, about 75% contribution is shared by hydrolytic enzymes.

In the post-genomic era, new enzyme technology developments are very crucial for market expansion (Wackett, 2011; Arbige et al., 2019). Megaliter fermenters are acting as microbial cell factories, engineered, and produced by rDNA technology and gene editing, that transform renewable carbon feedstocks into industrial products at a commodity scale. Programs that once took years to commercialize can now, in many circumstances, be finished in less than a year due to these new strain development methods.

The rising use of powerful enzymes is intended to improve human health, food, materials, animals, and agriculture (Jullesson et al., 2015; Arbige et al., 2019). These developments made pullulanase commercially available in the market but still it is limited. Among them the recombinant type I pullulanase Promozyme^®^ D2 (Novozyme) and Optimax^®^ L-1000 (DuPont Genencor^®^ Science), were derived from *Bacillus acidopullulyticus* and *Bacillus deramificans*, respectively. Other enzymes on the market include PU-799 from *Bacillus licheniformis* (Boli Bioproducts) and PUL2 from *Bacillus subtilis* (Sunsonzymes), both of which are produced in China (Kahar et al., 2022). In the patent search by using https://www.lens.org/we found 15,659 patents on pullulanase but still, it lacks its wide industrial application and market availability due to its higher production costs.

## 7 Industrial applications of pullulanase

Pullulanase has wide applications in various sectors such as Pharmaceuticals, baking, cyclodextrin production, etc. (Ray and Rosell, 2017). This enzyme has gained high importance in the production of sugar syrups and the preparation of resistant starch dental plaque control agents (Machida et al., 1986; Zhang and Jin, 2011).

### 7.1 Starch processing industry

Pullulanase has high demand in the starch processing industry for the manufacturing of maltotriose, maltotetraose, fructose, panose, isopanose, and glucose syrups. The production cost of glucose and maltose has been reduced because of an increase in their yield (Jensen and Norman, 1984). These groups of enzymes completely hydrolyze starch. Pullulanase along with amylase enhances the saccharification process and increases the quality of syrups produced by the enzymes. It is also used as an anti-staling agent in the food processing industry (Modderman and Foley, 1995). Biofuel-based industries using pullulanase to produce ethanol (Ramdas Malakar and Malviya, 2010).

### 7.2 Saccharification

The conversion of polysaccharides into simple sugars is called saccharification. Traditionally this process was achieved by the acid method but in recent days it is achieved by enzymatic hydrolysis. Pullulanase is preferably use as starch debranching enzyme in the saccharification process as compared to other enzymes to produce high-glucose or high maltose (Prongjit et al., 2022). It is mostly used in combination with *β*-amylase or glucoamylase (Yang et al., 2015; Li et al., 2021). HL12Pul collaborated with RSD -amylase HL11Amy to promote raw cassava starch saccharification, resulting in a 2.9-fold rise in reducing sugar results compared to HL11Amy alone (Prongjit et al., 2022).

### 7.3 High-maltose corn syrup

Pullulanase is used in the corn starch processing industry to obtain mild sweetness-based High fructose maltose syrup (HFMS) (Hii et al., 2012). They were also observed to tolerate a wider temperature and pH ranges, for instance, PulA-N3 (Niu et al., 2022) further enhancing the overall efficiency of the process.

HFMS is used in the manufacturing of high-quality candy and ice cream. The pharmaceutical industry is more focused on pure maltose which may be used as an alternative for D-glucose for intravenous feeding. Crystalline maltitol is produced from pure maltose (Varzakas et al., 2012).

### 7.4 High-fructose corn syrup (HFCS)

HFCS is a high-quality clean-tasting sweetener. The glucose isomerase is used to convert high glucose syrup (DE95-96) to HFCS. Here, pullulanase is commonly used for corn refining and debranching of high dextrose level into desirable levels (Helstad, 2019). A very high value of DE is essentially required for the manufacturing of crystalline glucose. HFCS is 1.2–1.8 times sweeter than sucrose but gives less energy than sucrose. It is mainly used in diabetic food formulation, as it is metabolized in absence of insulin (Henrissat and Davies, 1997; Cantarel et al., 2009).

### 7.5 Detergent

Enzymes have gained application in the detergent industry to remove starch under alkaline conditions (Upadek and Kttwitz, 1997). Because of the alkaline atmosphere of laundry detergents, only pullulanases (such as, pullulanase type II) with detergent-resistant, alkali-stable, and alkali-active capabilities are suitable for the detergent companies (Huang, et al., 2020). They are highly effective when used in combination with alkaline amylases (Ito et al., 1998). Another study that supported the application is the demonstration of the highest detergency value (R) and rate of detergency value (P) of pullulanase type I from *B. megaterium* Y103 when mixed with BlueMoon the commercial laundry detergent (Wu et al., 2022; Al-Mamoori et al., 2023).

### 7.6 Bioethanol

The agro-waste biomass can be used to convert into biofuel by using enzymes like pullulanase and amyloglucosidase (Nair et al., 2017). These enzymes convert the polysaccharides of these agro-wastes into reducing sugars which are further converted into alcohol by yeast. Due to the high conversion efficiency of pullulanase, they have high economic value in the biofuel industry. The most common example in this category is the conversion of duckweed into ethanol by using pullulanase (Xu et al., 2011; Yu et al., 2014).

### 7.7 Anti-staling agent in the baking industry

Globally starch modifying industries are used in several baking industries. Staling is a major problem faced by baking industries which changes both the chemical and physical attributes of the bread hence decreasing the quality of bakery products.

Retrogradation of starch is the major factor responsible for the staling of bakery products. The starch becomes insoluble from its soluble form thereby losing flexibility and becoming hard. When an appreciable amount of moisture is lost it becomes stale. To stop this process previously chemicals like potassium bromate and iodate were used for the treatment of flour.

Later, it was found that bromate is responsible for cancer hence it was banned globally. Similarly, iodate may be responsible for thyroid-associated disorders and sometimes cancer of the thyroid (EFSA NDA panel, 2014). The enzymes that have been extensively used as antistaling agents are amylase and glucoamylase (Else et al., 2013). Nowadays these enzymes have been replaced by pullulanase as an antistaling agent in the bakery industry. A thermostable pullulanase and α-amylase mixture (PersiPul1 and PersiAmy2) has been developed for usage in bread supplemented with quinoa protein that reduces the bread’s chewiness and hardness while increasing its specific volume, browning index and porosity. The presence of enzymes results in higher sensory scores for the functional bread. According to the findings, the new starch-degrading enzyme mixture is a potential option for increasing the physical and sensory properties of the antioxidant bread (Sadeghian Motahar et al., 2022). Pullulanase acts on maltodextrins to eliminate gumminess (Carroll et al., 1987).

### 7.8 Production of cyclodextrins (CDs)

Cyclodextrins (CDs) are generally used as stabilizers and solvents for poorly soluble drugs. They are potential industrial substrates for pharmaceuticals, cosmetics, agriculture, and in analytical chemistry. They are used in the manufacturing of cholesterol-free products (Kim et al., 2000).

During the conversion of starch into CDs, starch is liquefied by amylase and then cyclized by cyclodextrin glycosyltransferase (Van Der Maarel et al., 2002). But the major problem associated with this conversion is the blockage of the action of CGTase by amylopectin which was recently reported to be dealt with the use of pullulanase mutants (Li et al., 2021b). Moreover, Maltosyl-CDs and glucosyl–CDs are also produced by using Pullulanase (Rendleman, 1997).

### 7.9 Brewing low-calorie beer

The low content of carbohydrates or low calories in beer is obtained by adding pullulanase, amyloglucosidase or glucoamylase to the wort before or during fermentation (Mathews et al., 2001; Pati and Samantaray, 2022). These enzymes hydrolyze non fermentable carbohydrates due to the hydrolysis of branched α -1,6 glycosidic bonds of starch into fermentable sugar and reduce the calorie and alcohol content in beer (Blanco et al., 2014). Thereby, improving the yield and quality of the beer (Kłosowski et al., 2010; Prakash et al., 2012).

### 7.10 Food gums

Natural gums are used extensively in various food industries as stabilizing agents, emulsifying agents, thickening agents, and gelling agents. These gums have also found application as clarifying agents in beverage industries. Locust bean gums (cargo gum) are the most common example in this category. Carob seeds are used to extract galactomannan gum. The major problem associated with the extraction of this gum is its cost because the extraction process is very difficult. This problem can be removed by treating gaur galactomannan to remove galactose residue to obtain modified galactomannans with improving rheological properties like elasticity and viscosity (Shobha and Tharanathan, 2009).

### 7.11 Resistant starch (RS)

It is one of the most significant components of complete food fiber. RS has been used as a replacement in gluten-free wheat-based products. They are also used as prebiotics to promote the growth of probiotic bacteria. Fermentation of RS decreases the pH, ammonia, and phenols in the intestine. Moreover, it also has anti-carcinogenic and anti-inflammatory activities. It is fermented by colon microflora and produces smaller fatty acids and is considered a functional food. RS is produced mainly by using the enzyme pullulanase and amylase (Nibha, 2002; Luis et al., 2018). The debranching effect of pullulanase is responsible for the production of resistant starch from maize, kidney beans, elephant foot yam, and potato (Reddy et al., 2013; Reddy et al., 2014; Reddy et al., 2015).

### 7.12 Fruit juice clarification

**FIGURE 3:**
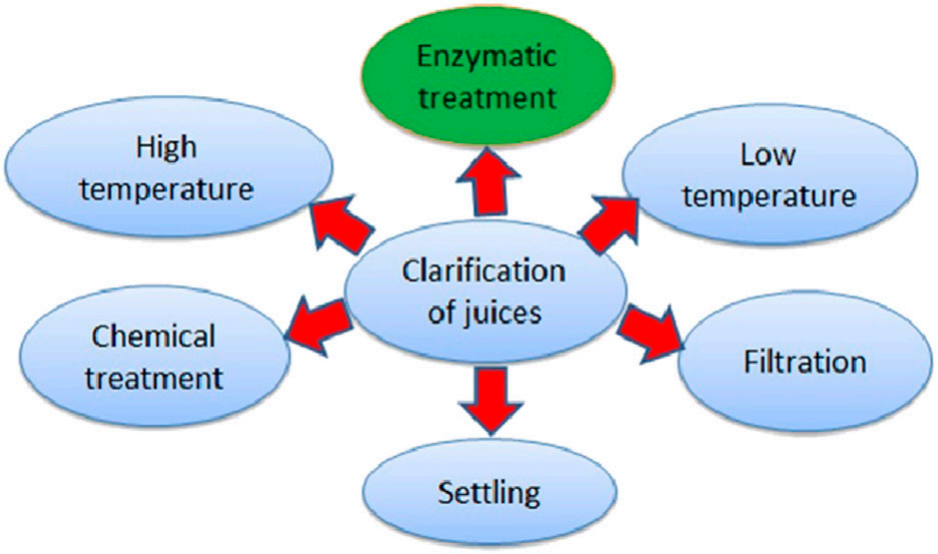
Different methods of juice clarification.

In clarification of fruit juices, all the suspended materials were removed to improve their appearance, quality, flavor, and yield. It is an important processing step during fruit juice production (Barbosa- Canova and Gould, 2000). It can achieve by both physical and chemical methods or by their combined effects (Figure 3).

From the above different processes, the enzymatic process using laccase, xylanase, cellulase, amylase, pectinases, etc., is the most common method including concentration of the enzyme, time and temperature of incubation, and the types of juice affects the efficiency of the fruit juice. An increasing in the concentration of enzyme gives the same results in half of the time and *vice versa*. On increasing the temperature (10°C) double the enzyme activity between 10°C–50°C. However, the enzyme may be inactivated at a higher temperature for a longer time of incubation. The other factors that affect the efficiency of juice clarification are the presence of enzyme inhibitors; unfavourable pH, high polyphenols, alcohol, and the presence of SO_2_ inactivating the enzymes.

In the treatment of fruit juice with enzymes, different parameters like TSS, turbidity, viscosity, pH, and phenol content are affected significantly (Sharma et al., 2017). Fruit juices showed a reduced viscosity due to a decrease in the TSS (Jiao et al., 2004). However, according to, Joshi et al. (2019); Joshi et al., 2011), the TSS of juices is increased on its enzymatic treatment due to the degradation of the matrix itself into small and soluble compounds, and on the extraction of more cellular liquids, more soluble compounds are recovered. TSS of the juice is increased by increasing the concentration or doses of the enzyme for the treatment of the juice. TSS and viscosity are inversely proportional, as the TSS increases the viscosity of the juice decreased equally (Tiruneh et al., 2021).

Acidity, total carbohydrates, ascorbic acid, and color intensity are the other factors that are increased in the clarification of fruit juice. However, pH showed a lowered value. Clarification of fruit juice approved as advantageous for the final product quality. It provides better consumer acceptance and nutritional properties to the final products. It also improves the shelf life of the treated juices (Joshi et al., 1991; Rinaldi et al., 2013). With these beneficial effects, it shows some limitations like bitterness in juice due to the accumulation of polyphenols, and low quality as compared to traditionally processed juice (Mieszczakowska-Frąc et al., 2012; Laaksonen et al., 2013).

## 8 Analytical techniques

### 8.1 Determination of average chain length

Pullulanase is used for the structural determination of glycogen and starch polysaccharides. Different enzymes are used for the determination of the chain length of polysaccharides like amylose, amylopectin, glycogen, etc. Pullulanase is one among them. Plant-sourced pullulanase (R-enzyme) along with β-amylase is used to determine the chain length of amylopectin, however, it cannot be used for the same in the case of glycogen because it does not attack glycogen. Substituting *Aerobacter* pullulanase with R-enzyme allows the determination of glycogen chain length (Lee and Wheelan, 1966).

### 8.2 Structural determination of polysaccharide

The release of maltose and malt triose from β-dextrins by *Aerobacter* pullulanase, and their measurement, have been used to calculate the proportion of chains. The structures of amylopectin (Lee and Whelan, 1966) and glycogen have subsequently been explored by debranching the polysaccharides with *Aerobacter* pullulanase and fractionating the unit chains on Sephadex G-50. Amylopectin structure was examined by making the β-amylase limit dextrin and treating it with R-enzyme. The release of maltose and maltotriose from β-dextrins by *Aerobacter* pullulanase, and their measurement have been used to calculate the proportion of A chains. Yeast isoamylase and *Aerobacter* pullulanase (Banks et al., 1974) have been used to prove that the anomalous (non-l-4) bonds known to be in amylose are α-1-6-glucosidic bonds.

### 8.3 Determination of enzyme action pattern

R-Enzyme was used to prove that the A chains in amylopectin, β-extrin are 2 and 3 glucose units in length, and the ϕ-dextrin are 4 units long*. Aerobacter* pullulanase was used to prove the same point for glycogen ϕ-dextrin*.* The structures of the oligosaccharide limit dextrin formed from amylopectin and glycogen by various α-amylases were determined in part by hydrolyzing them with R-enzyme (Whelan, 1960). Verhue and Hers (1966) and Brown and Brown (1966) studied liver and muscle branching enzymes by using pullulanase to split off A chains formed by the branching enzyme. Pullulanase was used to determine the unit-chain profiles of Q-enzyme (potato branching enzyme) synthesized amylopectin from amylose or, with phosphorylase, from a-glucose l-phosphate by fractionating the unit chain on Sephadex *G-50* followed by comparing the chain profile with the natural amylopectin.

## 9 Conclusion

Based on the review it can be concluded that very few reports are available on pullulanase production from native strains because of low yield issues. In line with the increasing demands for pullulanase, it has become important to search for novel pullulanase-producing microorganisms with high yields. Moreover, high production costs and low yield are major limitations in the industrial production of pullulanase enzymes. The production cost of pullulanase by using the solid-state fermentation (SSF) process can be minimized by selecting agro-industrial waste. Much agro-industrial waste such as sawdust, corn cobs, rice bran, green Gram husk, wheat straw, rice straw, wheat bran, soy hull, grape wine trimmings dust, sweet sorghum pulp, sugarcane bagasse, banana peel, palm oil mill waste, etc. can be used to produce this enzyme which can be achieved by using fungal strains producing pullulanase. Researchers should focus on fungal strains producing pullulanase for better yield and low production costs by using agro-waste. It will prove a better enzyme in different food processing industries and will surely reduce the cost of products.

## References

[B1] Ahmad Z. Butt M. S. Riaz M. (2013). Partial purification and characterization of xylanase produced from *Aspergillus niger* using wheat bran. Pak. J. Agric. Sci. 50 (3), 433–437.

[B2] Akassou M. Groleau D. (2019). Advances and challenges in the production of extracellular thermoduric pullulanases by wild-type and recombinant microorganisms: A review. Crit. Rev. Biotechnol. 1, 337–350. 10.1080/07388551.2019.1566202 30700157

[B3] Al-Mamoori Z. Z. Embaby A. M. Hussein A. Mahmoud H. E. (2023). A molecular study on recombinant pullulanase type I from Metabacillus indicus. Amb. Express 13 (1), 40. 10.1186/s13568-023-01545-8 37119334 PMC10148936

[B4] Ali G. Dulong V. Gasmi S. N. Rihouey C. Picton L. Le Cerf D. (2015). Covalent immobilization of pullulanase on alginate and study of its hydrolysis of pullulan. Biotechnol. Progr. 31 (4), 883–889. 10.1002/btpr.2093 25919860

[B5] Aoki H. Sakano Y. (1997). Molecular cloning and heterologous expression of the isopullulanase gene from *Aspergillus niger* ATCC 9642. Biochem. J. 323 (3), 757–764. 10.1042/bj3230757 9169610 PMC1218380

[B6] Ara K. Saeki K. Igarashi K. Takaiwa M. Uemura T. Hagihara H. (1995). Purification and characterization of an alkaline amylopullulanase with both α-1,4 and α-1,6 hydrolytic activity from alkalophilic Bacillus sp. KSM-1378. Biochim. Biophys. Acta, Gen. Subj. 1243 (3), 315–324. 10.1016/0304-4165(94)00148-q 7727505

[B7] Arbige M. V. Shetty J. K. Chotani G. K. (2019). Industrial enzymology: The next chapter. Trends Biotechnol. 37, 1355–1366. 10.1016/j.tibtech.2019.09.010 31679826

[B8] Archana A. Satyanarayana T. (1997). Xylanase production by thermophilic *Bacilluslicheniformis* A99 in solid-state fermentation. Enzyme Microb. Technol. 21 (1), 12–17. 10.1016/s0141-0229(96)00207-4

[B9] Armenta S. Moreno-Mendieta S. Sánchez-Cuapio Z. Sánchez S. Rodríguez-Sanoja R. (2017). Advances in molecular engineering of carbohydrate-binding modules. Proteins Struct. Funct. bioinform. 85, 1602–1617. 10.1002/prot.25327 28547780

[B10] Babbar N. Oberoi H. S. (2014). “Potential of agro-residues as sources of bioactive compounds,” in Biotransformation of waste biomass into high value biochemicals (New York, NY: Springer), 261–295.

[B11] Babu K. R. Satyanarayana T. (1995). α-Amylase production by thermophilic Bacillus coagulans in solid state fermentation. Process Biochem. 30 (4), 305–309. 10.1016/0032-9592(95)87038-5

[B12] Banerjee S. Ghosh U. (2017). Production and characterization of glucoamylase by. Aspergillus Niger. Appl. Food Biotechnol. 4 (1), 19–26. 10.22037/afb.v4i1.13261

[B13] Banks W. Greewood C. T. Muir D. D. (1974). Studies on starches of high amylose content. Part 17. A review of current concepts. Starch‐Stärke 26 (9), 289–300. 10.1002/star.19740260902

[B14] Barbosa-Cánova G. V. Gould G. W. (2000). Food preservation technologies: Innovations in food processing. Lancaster, Pennsylvania, USA: Technomic Publishing Co., Inc., 123–148.

[B15] Beaulieu L. Groleau D. Miguez C. B. Jetté J. F. Aomari H. Subirade M. (2005). Production of pediocin PA-1 in the methylotrophic yeast Pichia pastoris reveals unexpected inhibition of its biological activity due to the presence of collagen-like material. Protein Expr. Purif. 43 (2), 111–125. 10.1016/j.pep.2005.05.012 16023368

[B16] Bertoldo C. Antranikian G. (2002). Starch-hydrolyzing enzymes from thermophilic archaea and bacteria. Curr. Opin. Chem. Biol. 6 (2), 151–160. 10.1016/S1367-5931(02)00311-3 12038998

[B17] Bertoldo C. Duffner F. Jorgensen P. L. Antranikian G. (1999). Pullulanase type I from *fervidobacteriumpennavorans* Ven5: Cloning, sequencing, and expression of the gene and biochemical characterization of the recombinant enzyme. Appl. Environ. Microbiol. 65 (5), 2084–2091. 10.1128/aem.65.5.2084-2091.1999 10224005 PMC91302

[B18] Blazeck J. Garg R. Reed B. Alper H. S. (2012). Controlling promoter strength and regulation in *Saccharomyces cerevisiae* using synthetic hybrid promoters. Biotechnol. Bioeng. 109 (11), 2884–2895. 10.1002/bit.24552 22565375

[B19] Bodjrenou D. M. Li X. Chen W. Zhang Y. Zheng B. Zeng H. (2022). Effect of pullulanase debranching time combined with autoclaving on the structural, physicochemical properties, and *in vitro* digestibility of purple sweet potato starch. Foods 11 (23), 3779. 10.3390/foods11233779 36496587 PMC9740074

[B195] Blanco C. A. Caballero I. Barrios R. Rojas A. (2014). Innovations in the brewing industry: light beer. Int. J. Food Sci. Nutr. 65 (6), 655–660. 10.3109/09637486.2014.893285 24601667

[B20] Brown B. I. Brown D. H. (1966). Lack of an alpha-1, 4-glucan: alpha-1, 4-glucan 6-glycosyl transferase in a case of type IV glycogenosis. Proc. Natl. Acad. Sci. U. S. A. 56 (2), 725–729. 10.1073/pnas.56.2.725 5229990 PMC224432

[B21] Cantarel B. L. Coutinho P. M. Rancurel C. Bernard T. Lombard V. Henrissat B. (2009). The carbohydrate-active EnZymes database (CAZy): An expert resource for glycogenomics. Nucleic Acids Res. 37 (Suppl. l_1), D233–D238. 10.1093/nar/gkn663 18838391 PMC2686590

[B22] Carroll J. O. Boyce C. O. Wong T. M. (1987). U.S. Patent No. 4,654,216. Washington, DC: U.S. Patent and Trademark Office.

[B23] Chang M. Chu X. Lv J. Li Q. Tian J. Wu N. (2016). Improving the thermostability of acidic pullulanase from *Bacillus naganoensis* by rational design. PLoS ONE 11, e0165006. 10.1371/journal.pone.0165006 27764201 PMC5072709

[B24] Chen A. N. Xu T. T. Ge Y. Wang L. Y. Tang W. J. Li S. (2019). Hydrogen-bond-based protein engineering for the acidic adaptation of *Bacillus acidopullulyticus* pullulanase. Enzyme Microb. Technol. 124, 79–83. 10.1016/j.enzmictec.2019.01.010 30797482

[B25] Chen M. Zhang J. Wang J. Lin L. Wei W. Shen Y. (2022). A type I pullulanase from *geobacillussubterraneus*: Functional expression in *Escherichia coli*, enzyme characterization, truncation, and application. Starch‐Stärke 74 (11-12), 2200044. 10.1002/star.202200044

[B26] Choi J. M. Han S. S. Kim H. S. (2015). Industrial applications of enzyme biocatalysis: Current status and future aspects. Biotechnol. Adv. 33 (7), 1443–1454. 10.1016/j.biotechadv.2015.02.014 25747291

[B27] Coleman R. D. (1993). in Genetics and molecular biology of anaerobic bacteria (New York, NY: Springer), 640–653.

[B28] Dakhmouche Djekrif S. Bennamoun L. Labbani F. Z. K. Ait Kaki A. Nouadri T. Pauss A. (2021). An alkalothermophilic amylopullulanase from the yeast *Clavispora lusitaniae* ABS7: Purification, characterization and potential application in laundry detergent. Catalysts 11 (12), 1438. 10.3390/catal11121438

[B29] Desgranges C. Durand A. (1990). Effect of pCO2 on growth, conidiation, and enzyme production in solid-state culture on *Aspergillus niger* and *Trichoderma viride* TS. Enzyme Microb. Technol. 12 (7), 546–551. 10.1016/0141-0229(90)90073-y

[B30] Dessouki A. M. Issa G. I. Atia K. S. (2001). Pullulanase immobilization on natural and synthetic polymers. J. Chem.Technol. Biotechnol. 76 (7), 70–706. 10.1002/jctb.430

[B31] Domań-Pytka M. Bardowski J. (2004). Pullulan degrading enzymes of bacterial origin. Crit. Rev. Microbiol. 30 (2), 107–121. 10.1080/10408410490435115 15239382

[B32] Duan X. Chen J. Wu J. (2013). Improving the thermostability and catalytic efficiency of *Bacillus deramificans* pullulanase by site-directed mutagenesis. Appl. Environ. Microbiol. 79, 4072–4077. 10.1128/aem.00457-13 23624477 PMC3697558

[B33] Duffner F. Bertoldo C. Andersen J. T. Wagner K. Antranikian G. (2000). A new thermoactivepullulanase from *desulfurococcusmucosus*: Cloning, sequencing, purification, and characterization of the recombinant enzyme after expression in *Bacillus subtilis* . J. Bacteriol. 182 (22), 6331–6338. 10.1128/JB.182.22.6331-6338.2000 11053376 PMC94778

[B34] EFSA NDA Panel (EFSA Panel on Dietetic Products, Nutrition and Allergies) (2014). Scientific opinion on dietary reference values for selenium. EFSA J. 12, 3846. 10.2903/j.efsa.2014.3846 PMC13136995 42087966

[B35] Else A. J. Tronsmo K. M. Niemann L. A. Moonen J. H. E. (2013). U.S. Pat. Appl. no. 13/593, 317.

[B36] Fadel M. AbdEl-Halim S. Sharada H. Yehia A. Ammar M. (2020). Production of glucoamylase, α-amylase and cellulase by *Aspergillus oryzae* F-923 Cultivated on wheat bran under solid state fermentation. J. Adv. Biol. Biotechnol. 30, 8–22. 10.9734/jabb/2020/v23i430149

[B37] Gajdhane S. B. Bhagwat P. K. Dandge P. B. (2016). Statistical media optimization for enhanced production of α-galactosidase by a novel Rhizopus oryzae strain SUK. Biocatal. Agric. Biotechnol. 8, 301–309. 10.1016/j.bcab.2016.08.016

[B38] Gangadharan D. Sivaramakrishnan S. (2009). “Amylolytic enzymes,” in Biotechnology for agro-industrial residues utilisation (Dordrecht: Springer), 359–369.

[B39] Garcia‐Galan C. Berenguer‐Murcia Á. Fernandez‐Lafuente R. Rodrigues R. C. (2011). Potential of different enzyme immobilization strategies to improve enzyme performance. Adv. Synth. Catal. 353 (16), 2885–2904. 10.1002/adsc.201100534

[B40] Ghosh B. Ray R. R. (2011). Extra-cellular isoamylase production by *Rhizopus oryzae* in solid-state fermentation of agro wastes. Braz. Arch. Biol. Technol. 54, 867–876. 10.1590/s1516-89132011000500003

[B41] Gomes I. Gomes J. Steiner W. (2003). Highly thermostable amylase and pullulanase of the extreme thermophilic eubacterium *Rhodothermus marinus*: Production and partial characterization. Bioresour. Technol. 90 (2), 207–214. 10.1016/S0960-8524(03)00110-X 12895565

[B42] Gowthaman M. K. Krishna C. Moo-Young M. (2001). “Applied mycology and biotechnology,” in Agriculture and food productions. Editors Khachatourians G. G. Arora D. K. (Netherlands: Elsevier Science), Vol. 1, 305–352.

[B43] Guo J. Coker A. R. Wood S. P. Cooper J. B. Keegan R. M. Ahmad N. (2018). Structure and function of the type III pullulan hydrolase from*Thermococcus kodakarensis* . Thermococcuskodakarensis *Acta Crystallogr. Sect. D. Biol. Crystallogr.* 74 (4), 305–314. 10.1107/S2059798318001754 29652257

[B44] Hamidi M. Kennedy J. F. Khodaiyan F. Mousavi Z. Hosseini S. S. (2019). Production optimization, characterization and gene expression of pullulan from a new strain of *Aureobasidium pullulans* . Int. J. Biol. Macromol. 138, 725–735. 10.1016/j.ijbiomac.2019.07.123 31340178

[B45] Hanefeld U. Gardossi L. Magner E. (2009). Understanding enzyme immobilisation. Chem. Soc. Rev. 38 (2), 453–468. 10.1039/b711564b 19169460

[B46] Helstad S. (2019). “Corn sweeteners,” in Corn (USA: AACC International Press), 551–591.

[B47] Henrissat B. Davies G. (1997). Structural and sequence-based classification of glycoside hydrolases. Curr. Opin. Struct. Biol. 7 (5), 637–644. 10.1016/s0959-440x(97)80072-3 9345621

[B48] Hii S. L. Tan J. S. Ling T. C. Ariff A. B. (2012). Pullulanase: Role in starch hydrolysis and potential industrial applications. Enzyme Res. 1, 1–14. 10.1155/2012/921362 PMC3443597 22991654

[B49] Huang H. Lin Y. Wang G. Lin J. (2020). Gene cloning, expression and biochemical characterization of a new multi-domain, halotolerant and SDS-resistant alkaline pullulanase from Alkalibacterium sp. SL3. . *Process Biochem.* 96, 1–10. 10.1016/j.procbio.2020.05.019

[B50] Imanaka T. A. D. A. Y. U. K. I. Kuriki T. A. K. A. S. H. I. (1989). Pattern of action of *Bacillus stearothermophilus* neopullulanase on pullulan. J. Bacteriol. 171 (1), 369–374. 10.1128/jb.171.1.369-374.1989 2914851 PMC209598

[B51] Ishizaki Y. Taniguchi H. Maruyama Y. Nakamura M. (1983). Debranching enzymes of potato tubers (*Solanum tuberosum* l) II. purification of a pullulanase (R-enzyme) from potato tubers and comparison of its properties with those of the potato isoamylase. J. Jpn. Soc. Starch Sci. 30, 19–29. 10.5458/jag1972.30.19

[B52] Ito S. Kobayashi T. Ara K. Ozaki K. Kawai S. Hatada Y. (1998). Alkaline detergent enzymes from alkaliphiles: Enzymatic properties, genetics, and structures. Extremophiles 2 (3), 185–190. 10.1007/s007920050059 9783164

[B53] Iyer P. V. Ananthanarayan L. (2008). Enzyme stability and stabilization—Aqueous and non-aqueous environment. Process Biochem. 43 (10), 1019–1032. 10.1016/j.procbio.2008.06.004

[B54] Janeček Š. Majzlová K. Svensson B. MacGregor E. A. (2017). The starch-binding domain family CBM41—An *in silico* analysis of evolutionary relationships. Proteins Struct. Funct. bioinform. 85, 1480–1492. 10.1002/prot.25309 28425599

[B55] Jensen B. F. Norman B. E. (1984). *Bacillusacidopullulyticus*pullulanase: Application and regulatory aspects for use in the food industry. Process Biochem. 19 (4), 129–134.

[B56] Jiao B. Cassano A. Drioli E. (2004). Recent advances on membrane processes for the concentration of fruit juices: A review. J. Food. Eng. 63 (3), 303–324. 10.1016/j.jfoodeng.2003.08.003

[B57] Joshi V. K. Chauhan S. K. Lal B. B. (1991). Extraction of juices from peaches, plums and apricots by pectinolytic treatment. J. Food Sci. Technol. 28 (1), 64–65.

[B58] Joshi V. K. Parmar M. Rana N. (2011). Purification and characterization of pectinase produced from apple pomace and evaluation of its efficacy in fruit juice extraction and clarification. Indian J. Nat. Prod. Resour. 2 (2), 189–197.

[B59] Jòzef S. (2007). “The use of starch processing enzymes in the food industry,” in Industrial enzymes (Dordrecht: Springer), 19–34.

[B60] Juhas M. Reuß D. R. Zhu B. Commichau F. M. (2014). *Bacillus subtilis* and *Escherichia coli* essential genes and minimal cell factories after one decade of genome engineering. Microbiol 160 (11), 2341–2351. 10.1099/mic.0.079376-0 25092907

[B61] Jullesson D. David F. Pfleger B. Nielsen J. (2015). Impact of synthetic biology and metabolic engineering on industrial production of fine chemicals. Biotechnol. Adv. 33, 1395–1402. 10.1016/j.biotechadv.2015.02.011 25728067

[B62] Kahar U. M. Latif N. A. Amran S. I. Liew K. J. Goh K. M. (2022). A bibliometric analysis and review of pullulan-degrading enzymes—Past and current trends. Catalysts 12 (2), 143. 10.3390/catal12020143

[B63] Kang H. K. Jang J. H. Shim J. H. Park J. T. Kim Y. W. Park K. H. (2010). Efficient constitutive expression of thermostable 4-alpha-glucanotransferase in *Bacillussubtilis* using dual promoters. World J. Microbiol. Biotechnol. 26, 1915–1918. 10.1007/s11274-010-0351-5

[B64] Kashiwabara S. I. Ogawa S. Miyoshi N. Oda M. Suzuki Y. (1999). Three domains comprised in thermostable molecular weight 54,000 pullulanase of type I from Bacillus flavocaldarius KP1228. Biosci. Biotechnol. Biochem. 63 (10), 1736–1748. 10.1271/bbb.63.1736 10586502

[B65] Kaur H. Arora M. Bhatia S. Alam M. S. (2015). Optimization of α-amylase and glucoamylase production in solid state fermentation of deoiled rice bran (DRB) by. Rhizopusoryzae. Int. J. Pure Appl. Biosci. 3 (6), 49–256. 10.18782/2320-7051.2143

[B66] Khalaf A. K. Aldeen S. B. (2013). Optimum condition of pullulanase production by liquid state and solid state fermentation (SSF) method from Bacillus licheniforms (BS18). Iraq J. Sci. 54, 35–49.

[B67] Kim C. H. Nashiru O. Ko J. H. (1999). Purification and biochemical characterization of pullulanase type I from *Thermuscaldophilus* GK-24. FEMS Microbiol. Lett. 138 (2-3), 147–152. 10.1111/j.1574-6968.1996.tb08148.x 9026441

[B68] Kim J-H. Sunako M. Ono H. Murooka Y. Fukusaki E. Yamashita M. (2009). Characterization of the C-terminal truncated form of amylopullulanase from *Lactobacillus plantarum* L137. J. Biosci. Bioeng. 107, 124–129. 10.1016/j.jbiosc.2008.10.019 19217549

[B69] Kim J. H. Hwang B. Y. Roh J. Lee J. K. Kim K. Wong S. L. (2008). Camparison of P-aprE, P-amyE, and P-P43 promoter strength for beta-galactosidase and staphylokinase expression in *Bacillus subtilis* . Biotechnol. Bioprocess Eng. 13, 313–318. 10.1007/s12257-007-0102-0

[B70] Kim J. H. Kim M. R. Lee J. H. Lee J. W. Kim S. K. (2000). Production of high molecular weight pullulan by *Aureobasidium pullulans* using glucosamine. Biotechnol. Lett. 22 (12), 987–990. 10.1023/A:1005681019573

[B71] Kłosowski G. Mikulski D. Czupryński B. Kotarska K. (2010). Characterisation of fermentation of high-gravity maize mashes with the application of pullulanase, proteolytic enzymes and enzymes degrading non-starch polysaccharides. J. Biosci. Bioeng. 109 (5), 466–471. 10.1016/j.jbiosc.2009.10.024 20347769

[B72] Kong H. G. Choi K. H. Heo K. R. Lee K. Y. Lee H. J. Moon B. J. (2009). Generation of a constitutive green fluorescent protein expression construct to mark biocontrol bacteria using p43 promoter from *Bacillus subtilis* . *Plant Pathol.* J. 25, 136–141. 10.5423/Ppj.2009.25.2.136

[B73] Krishna C. (2005). Solid-state fermentation systems-an overview. Crit. Rev. Biotechnol. 25 (1), 1–30. 10.1080/07388550590925383 15999850

[B74] Kumar A. Kumari S. Dindhoria K. Manyapu V. Kumar R. (2022). “Efficient utilization and bioprocessing of agro-industrial waste,” in Sustainable agriculture reviews 56: Bioconversion of food and agricultural waste into value-added materials (Cham: Springer International Publishing), 1–37.

[B75] Kumar V. Ahluwalia V. Saran S. Kumar J. Patel A. K. an Singhania R. R. (2020). Recent developments on solid-state fermentation for production of microbial secondary metabolites: Challenges and solutions. Bioresour. Technol. 19, 124566. 10.1016/j.biortech.2020.124566 33390315

[B76] Kunamneni A. Permaul K. Singh S. (2005). Amylase production in solid state fermentation by the thermophilic fungus *Thermomyceslanuginosus* . J. Biosci. Bioeng. 100 (2), 168–171. 10.1263/jbb.100.168 16198259

[B77] Kuriki T. Okada S. Imanaka T. (1988). New type of pullulanase from *Bacillus stearothermophilus* and molecular cloning and expression of the gene in *Bacillussubtilis* . J. Bacteriol. 170 (4), 1554–1559. 10.1128/jb.170.4.1554-1559.1988 3127377 PMC211001

[B78] Laaksonen O. Mäkilä L. Tahvonen R. Kallio H. Yang B. (2013). Sensory quality and compositional characteristics of blackcurrant juices produced by different processes. Food Chem. 138 (4), 2421–2429. 10.1016/j.foodchem.2012.12.035 23497904

[B79] Lam C. M. Diez M. S. Godinho M. dos Santos V. A. M. (2012). Programmable bacterial catalysis–designing cells for biosynthesis of value-added compounds. FEBS Lett. 586 (15), 2184–2190. 10.1016/j.febslet.2012.02.030 22710181

[B80] Leathers T. D. (2003). Biotechnological production and applications of pullulan. Appl. Microbiol. Biotechnol. 62 (5), 468–473. 10.1007/s00253-003-1386-4 12830329

[B82] Lee E. Y. C. Whelan W. J. (1966). Enzymic methods for the microdetermination of glycogen and amylopectin, and their unit-chain lengths. *Arch. Biochem. Biophys*16 116, 162–167. 10.1016/0003-9861(66)90024-5 5961834

[B83] Lévêque E. Janeček Š. Haye B. an Belarbi A. (2000). Thermophilic archaeal amylolytic enzymes. Enzyme Microb. Technol. 26 (1), 3–14. 10.1016/s0141-0229(99)00142-8

[B84] Li C. Kong H. Yang Q. Gu Z. Ban X. Cheng L. (2021). A temperature‐mediated two‐step saccharification process enhances maltose yield from high‐concentration maltodextrin solutions. J. Sci. Food Agric. 101 (9), 3742–3748. 10.1002/jsfa.11005 33301206

[B85] Li S. Yang X. Yang S. Zhu M. Wang X. (2012). Technology prospecting on enzymes: Application, marketing and engineering. *Comput. Struct. Biotechnol. J* **.** 2 (3), e201209017. 10.5936/csbj.201209017 24688658 PMC3962110

[B86] Li X. Ji H. Bai Y. Jin Z. (2021b). Development of pullulanase mutants to enhance starch substrate utilization for efficient production of β-CD. Int. J. Biol. Macromol. 168, 640–648. 10.1016/j.ijbiomac.2020.11.120 33220368

[B87] Liebl W. Stemplinger I. Ruile P. (1997). Properties and gene structure of the *Thermotoga maritima* alpha-amylase AmyA, a putative lipoprotein of a hyperthermophilic bacterium. J. Bacteriol. 79 (3), 941–948. 10.1128/jb.179.3.941-948.1997 PMC178779 9006052

[B88] Ling H. Chuan L. Rosfarizan M. Ariff A. B. (2009). Characterization of pullulanase type II from *Bacillus cereus* H15. Am. J. Biochem. Biotechnol. 5 (4), 170–179. 10.3844/ajbbsp.2009.170.179

[B89] Liu X. Kokare C. (2023). “Microbial enzymes of use in industry,” in Biotechnology of microbial enzymes (Germany: Academic Press), 405–444.

[B90] Long J. Li X. Liu X. Jin Z. Xie Z. Xu X. (2021). Preparation of streptavidin-coated magnetic nanoparticles for specific immobilization of enzymes with high activity and enhanced stability. Ind. Eng. Chem. Res. 60 (4), 1542–1552. 10.1021/acs.iecr.0c03281

[B91] Lonsane B. K. Saucedo-Castaneda S. Raimbault M. Roussos S. Viniegra-Gonzalez G. G. Ghildyal N. P. (1992). Scale-up strategies for solid state fermentation systems. Process Biochem. 27, 259–273. 10.1016/0032-9592(92)85011-p

[B92] López-Gómez J. P. Venus J. (2021). Potential role of sequential solid-state and submerged-liquid fermentations in a circular bioeconomy. Fermentation 7 (2), 76. 10.3390/fermentation7020076

[B93] Luis A. S. Briggs J. Zhang X. Farnell B. Ndeh D. Labourel A. (2018). Dietary pectic glycans are degraded by coordinated enzyme pathways in human colonic Bacteroides. Nat. Microbiol. 3 (2), 210–219. 10.1038/s41564-017-0079-1 29255254 PMC5784806

[B94] Machida Y. Fukui F. Komoto T. (1991). Use of oligosaccharides for promoting the proliferation of Bifidobacteria. EP Patent 0242459 B1

[B95] Martins S. Mussatto S. I. Martínez-Avila G. Montañez-Saenz J. Aguilar C. N. Teixeira J. A. (2011). Bioactive phenolic compounds: Production and extraction by solid-state fermentation. A review. Biotechnol. Adv. 29 (3), 365–373. 10.1016/j.biotechadv.2011.01.008 21291993

[B96] Mateo C. Palomo J. M. Fernandez-Lorente G. Guisan J. M. Fernandez-Lafuente R. (2007). Improvement of enzyme activity, stability and selectivity via immobilization techniques. Enzyme Microb. Technol. 40 (6), 1451–1463. 10.1016/j.enzmictec.2007.01.018

[B97] Mathupala S. P. Lowe S. E. Podkovyrov S. M. Zeikus J. G. (1993). Sequencing of the amylopullulanase (apu) gene of *Thermoanaerobacterethanolicus* 39E, and identification of the active site by site-directed mutagenesis. J. Biol. Chem. 268 (22), 16332–16344. 10.1016/s0021-9258(19)85426-1 8344920

[B98] Matthews S. L. Byrne H. Hennigan G. P. (2001). Preparation of a low carbohydrate beer by mashing at high temperature with glucoamylase. J. Inst. Brew. 107 (3), 185–194. 10.1002/j.2050-0416.2001.tb00090.x

[B99] Meng F. Zhu X. Nie T. Lu F. Bie X. Lu Y. (2018). Enhanced expression of pullulanase in *Bacillus subtilis* by new strong promoters mined from transcriptome data, both alone and in combination. Front. Microbiol. 9, 2635. 10.3389/fmicb.2018.02635 30450090 PMC6224515

[B100] Mesbah N. M. Wiegel J. (2018). Biochemical characterization of halophilic, alkalithermophilic amylopullulanase PulD7 and truncated amylopullulanases PulD7ΔN and PulD7ΔC. Int. J. Biol. Macromol. 111, 632–638. 10.1016/j.ijbiomac.2018.01.069 29343451

[B101] Miao M. Jiang B. Zhang T. (2009). Effect of pullulanase debranching and recrystallization on structure and digestibility of waxy maize starch. Carbohydr. Polym. 76 (2), 214–221. 10.1016/j.carbpol.2008.10.007

[B102] Mieszczakowska-Frąc M. Markowski J. Zbrzeźniak M. &Płocharski W. (2012). Impact of enzyme on quality of blackcurrant and plum juices. LWT-Food Sci. Technol. 49 (2), 251–256. 10.1016/j.lwt.2011.12.034

[B103] Modderman J. P. Foley H. H. (1995). Safety evaluation of pullulanase enzyme preparation derived from Bacillus licheniformis containing the pullulanase gene from *Bacillusderamificans* . Regul. Toxicol. Pharmacol. 21 (3), 375–381. 10.1006/rtph.1995.1052 7480891

[B104] Møller M. S. Windahl M. S. Sim L. Bøjstrup M. Abou Hachem M. Hindsgaul O. (2015). Oligosaccharide and substrate binding in the starch debranching enzyme barley limit dextrinase. J. Mol. Biol. 427, 1263–1277. 10.1016/j.jmb.2014.12.019 25562209

[B105] Mrudula S. Reddy G. Seenayya G. (2011). Effect of substrate and culture conditions on the production of amylase and pullulanase by thermophilic *Clostridium thermosulforegenes* SVM17 in solid state fermentation. Malayas. J. Microbiol. 7, 19–25.

[B106] Naik B. Goyal S. K. Tripathi A. D. Kumar V. (2021). Exploring the diversity of endophytic fungi and screening for their pullulanase-producing capabilities. J. Genet. Eng. Biotechnol. 19 (1), 110–10. 10.1186/s43141-021-00208-0 34324093 PMC8322383

[B107] Naik B. Goyal S. K. Tripathi A. D. Kumar V. (2019). Screening of agro-industrial waste and physical factors for the optimum production of pullulanase in solid-state fermentation from endophytic *Aspergillus* sp. Biocatal. Agric. Biotechnol. 22, 101423. 10.1016/j.bcab.2019.101423

[B108] Nair R. B. Lennartsson P. R. andTaherzadeh M. J. (2017). “Bioethanol production from agricultural and municipal wastes,” in Current developments in biotechnology and bioengineering (Germany: Elsevier), 157–190.

[B109] Niba L. L. (2002). Resistant starch: A potential functional food ingredient. Nutr. Food Sci. 32 (2), 62–67. 10.1108/00346650210416985

[B110] Niehaus F. Peters A. Groudieva T. Antranikian G. (2000). Cloning, expression and biochemical characterization of a unique thermostable pullulan-hydrolysing enzyme from the hyperthermophilic archaeon *Thermococcus aggregans* . FEMS Microbiol. Lett. 15, 223–229. 10.1111/j.1574-6968.2000.tb09290 11034283

[B111] Nisha M. Satyanarayana T. (2016). Characteristics, protein engineering and applications of microbial thermostable pullulanases and pullulan hydrolases. Appl. Microbiol. Biotechnol. 100, 5661–5679. 10.1007/s00253-016-7572-y 27142298

[B112] Nisha M. Satyanarayana T. (2015). The role of N1 domain on the activity, stability, substrate specificity and raw starch binding of amylopullulanase of the extreme thermophile *Geobacillus thermoleovorans* . Appl. Microbiol. Biotechnol. 99, 5461–5474. 10.1007/s00253-014-6345-8 25573470

[B113] Niu D. Cong H. Zhang Y. Mchunu N. P. Wang Z. X. (2022). Pullulanase with high temperature and low pH optima improved starch saccharification efficiency. Sci. Rep. 12 (1), 21942. 10.1038/s41598-022-26410-9 36536070 PMC9763405

[B114] Pandey A. K. Sirohi R. Gaur V. K. Pandey A. (2021). Production and applications of pullulan. Biomass, Biofuels, Biochem., 165–221. 10.1016/b978-0-12-821888-4.00018-6

[B115] Pandey A. Soccol C. R. Mitchell D. (2000). New developments in solid state fermentation: I-Bioprocesses and products. Process Biochem. 35 (10), 1153–1169. 10.1016/s0032-9592(00)00152-7

[B116] Pang B. Zhou L. Cui W. Liu Z. Zhou Z. (2020). Improvement of the thermostability and activity of pullulanase from *Anoxybacillus* sp. WB42. Appl. Biochem. Biotechnol. 15, 942–954. 10.1007/s12010-020-03249-2 31939086

[B117] Park S. H. Na Y. Kim J. Dal Kang S. Park K. H. (2018). Properties and applications of starch modifying enzymes for use in the baking industry. Food Sci. Biotechnol. 27 (2), 299–312. 10.1007/s10068-017-0261-5 30263753 PMC6049653

[B118] Pati S. Samantaray D. P. (2022). “Enzymes in brewing and wine industries,” in Novel food grade enzymes: Applications in food processing and preservation industries (Singapore: Springer Nature Singapore), 165–181.

[B119] Paul J. S. Gupta N. Beliya E. Tiwari S. Jadhav S. K. (2021). Aspects and recent trends in microbial α-amylase: A review. Appl. Biochem. Biotechnol. 193, 2649–2698. 10.1007/s12010-021-03546-4 33715051

[B120] Poliakoff M. Licence P. (2007). Sustainable technology: Green chemistry. Nature*450* 450 (7171), 810–812. 10.1038/450810a 18064000

[B121] Prabhu N. Singh M. V. Karunakaran S. Kaliappan C Gajendran T. T. G. (2018). Production and purification of extracellular pullulanase by *Klebsilla aerogenes* NCIM 2239. Afr. J. Biotechnol. 17 (14), 486–494. 10.5897/AJB2017.15915

[B122] Prakash N. Gupta S. Ansari M. Khan Z. A. Suneetha V. (2012). Production of economically important products by the use ofpullulanase enzyme. Int. J. Sci. Innov. Discov. 2 (2), 266–273.

[B123] Prongjit D. Lekakarn H. Bunterngsook B. Aiewviriyasakul K. Sritusnee W. Arunrattanamook N. (2022). In-depth characterization of debranching type i pullulanase from *Priestia koreensis* HL12 as potential biocatalyst for starch saccharification and modification. Catalysts 12 (9), 1014. 10.3390/catal12091014

[B124] Ramdas Malakar D. Malviya S. N. Tiwari A. (2010). Pullulanase: A potential enzyme for industrial application. Int. J. Biomed. Res. 1 (2), 10–20. 10.7439/ijbr.v1i2.53

[B125] Ravindran R. Hassan S. S. Williams G. A. Jaiswal A. K. (2018). A review on bioconversion of agro-industrial wastes to industrially important enzymes. Bioengineering 5 (4), 93. 10.3390/bioengineering5040093 30373279 PMC6316327

[B126] Ray R. C. Rosell C. M. (2017). Microbial enzyme technology in food applications. New York: CRC Press.

[B127] Reddy C. K. Haripriya S. Mohamed A. N. Suriya M. (2014). Preparation and characterization of resistant starch III from elephant foot yam (*Amorphophallus paeonifolius*) starch. Food Chem. 155, 38–44. 10.1016/j.foodchem.2014.01.023 24594151

[B128] Reddy C. K. Pramila S. Haripriya S. (2015). Pasting, textural and thermal properties of resistant starch prepared from potato (*Solaumtuberosum*) starch using pullulanase enzyme. J. Food Sci. Technol. 52 (3), 1594–1601. 10.1007/s13197-013-1151-3 25745229 PMC4348285

[B129] Reddy C. K. Suriya M. Haripriya S. (2013). Physico-chemical and functional properties of Resistant starch prepared from red kidney beans (*Phaseolusvulgaris*. L) starch by enzymatic method. Carbohydr. Polym. 95 (1), 220–226. 10.1016/j.carbpol.2013.02.060 23618263

[B130] Reddy P. R. M. Reddy G. Seenayya G. (1999). Production of thermostable β-amylase and pullulanase by *Clostridium thermosulfurogenes* SV2 in solid-state fermentation: Screening of nutrients using Plackett-Burman design. Bioprocess Eng. 21 (2), 175–179. 10.1007/pl00009069

[B131] Rendleman J. A. Jr. (1997). Enhancement of cyclodextrin production through use of debranching enzymes. Biotechnol. Appl. Biochem. 26 (1), 51–61. 10.1111/j.1470-8744.1997.tb00446.x 9262003

[B132] Renz A. Schikora S. Schmid R. Kossmann J. Beck E. (1998). cDNA sequence and heterologous expression of monomeric spinach pullulanase: multiple isomeric forms arise from the same polypeptide. Biochem. J. 331, 937–945. 10.1042/bj3310937 9560325 PMC1219438

[B133] Rinaldi M. Caligiani A. Borgese R. Palla G. Barbanti D. Massini R. (2013). The effect of fruit processing and enzymatic treatments on pomegranate juice composition, antioxidant activity and polyphenols content. LWT-Food Sci. Technol. 53 (1), 355–359. 10.1016/j.lwt.2013.02.015

[B134] Rodrigues R. C. Ortiz C. Berenguer-Murcia Á. Torres R. Fernández-Lafuente R. (2013). Modifying enzyme activity and selectivity by immobilization. Chem. Soc. Rev. 42 (15), 6290–6307. 10.1039/c2cs35231a 23059445

[B135] Roodi F. Z. Aminzadeh S. Farrokhi N. Karkhane A. A. Haghbeen K. (2017). *Cohnella* amylopullulanases: Biochemical characterization of two recombinant thermophilic enzymes. PLoS One 12, e0175013. 10.1371/journal.pone.0175013 28394913 PMC5386253

[B136] Roy I. Gupta M. N. (2004). Hydrolysis of starch by a mixture of glucoamylase and pullulanase entrapped individually in calcium alginate beads. Enzyme Microb. Technol. 34 (1), 26–32. 10.1016/j.enzmictec.2003.07.001

[B137] Rüdiger A. Jorgensen P. L. Antranikian G. (1995). Isolation and characterization of a heat-stable pullulanase from the hyperthermophilic archaeon *Pyrococcuswoesei* after cloning and expression of its gene in *Escherichia coli* . Appl. Environ. Microbiol. 61 (2), 567–575. 10.1128/aem.61.2.567-575.1995 7574598 PMC167320

[B191] Rubilar O. Diez M. C. Gianfreda L. (2008). Transformation of chlorinated phenolic compounds by white rot fungi. Crit. Rev. Environ. Sci. Technol. 38, 227–268. 10.1080/10643380701413351

[B138] Sadeghian Motahar S. F. Salami M. Ariaeenejad S. Emam‐Djomeh Z. Sheykh Abdollahzadeh Mamaghani A. Kavousi K. (2022). Synergistic effect of metagenome‐derived starch‐degrading enzymes on quality of functional bread with antioxidant activity. Starch‐Stärke 74 (1-2), 2100098. 10.1002/star.202100098

[B139] Sakano Y. Masuda N. Kobayashi T. (1971). Hydrolysis of pullulan by a novel enzyme from *Aspergillus niger* . Agric. Biol. Chem. 35 (6), 971–973. 10.1080/00021369.1971.10860023

[B140] Salgado-Bautista D. Volke-Sepúlveda T. Figueroa-Martínez F. Carrasco-Navarro U. Chagolla-López A. Favela-Torres E. (2020). Solid-state fermentation increases secretome complexity in *Aspergillus brasiliensis* . Fungal Biol. 124 (8), 723–734. 10.1016/j.funbio.2020.04.006 32690254

[B141] Šelo G. Planinić M. Tišma M. Tomas S. KocevaKomlenić D. Bucić-Kojić A. (2021). A comprehensive review on valorization of agro-food industrial residues by solid-state fermentation. Foods 10 (5), 927. 10.3390/foods10050927 33922545 PMC8146281

[B142] Sharma H. P. Patel H. Sugandha P. (2017). Enzymatic added extraction and clarification of fruit juices–A review. Crit. Rev. Food Sci. Nutr. 13 57 (6), 1215–1227. 10.1080/10408398.2014.977434 26731188

[B143] Sharma R. Oberoi H. S. Dhillon G. S. (2016). Agro-industrial wastes as feedstock for enzyme production. Massachusetts: Academic Press, 23–59.

[B144] Sharma V. Tsai M. L. Nargotra P. Chen C. W. Kuo C. H. Sun P. P. (2022). Agro-industrial food waste as a low-cost substrate for sustainable production of industrial enzymes: A critical review. Catalysts 12 (11), 1373. 10.3390/catal12111373

[B145] Shingel K. I. (2004). Current knowledge on biosynthesis, biological activity, and chemical modification of the exopolysaccharide, pullulan. Carbohydr. Res. 339 (3), 447–460. 10.1016/j.carres.2003.10.034 15013381

[B146] Shobha M. S. Tharanathan R. N. (2009). Rheological behaviour of pullulanase-treated guar galactomannan on co-gelation with xanthan. Food Hydrocoll. 23 (3), 749–754. 10.1016/j.foodhyd.2008.04.006

[B147] Singh R. S. Saini G. K. Kennedy J. F. (2010). Covalent immobilization and thermodynamic characterization of pullulanase for the hydrolysis of pullulan in batch system. Carbohydr. Polym. 81 (2), 252–259. 10.1016/j.carbpol.2010.02.027

[B148] Singh R. S. Saini G. K. Kennedy J. F. (2008). Pullulan: Microbial sources, production and applications. Carbohydr. Polym. 73 (4), 515–531. 10.1016/j.carbpol.2008.01.003 26048217

[B149] Singh S. Cheema S. K. Kaur B. Mann N. K. (2013). Sorbitol: An enhancer of growth and alpha-amylase production for *Aspergillus fumigatus* NTCC1222 using wheat bran as substrate. Int. J. Biotechnol. Bioeng. Res. 4 (6), 555–560.

[B150] Song Y. Fu G. Dong H. Li J. Du Y. Zhang D. (2017). High-efficiency secretion of β-mannanase in Bacillus subtilis through protein synthesis and secretion optimization. J. Agric. Food Chem. 65 (12), 2540–2548. 10.1021/acs.jafc.6b05528 28262014

[B192] Soares I. Távora Z. Barcelos R. P. Baroni S. (2012). “Microorganism-produced enzymes in the food industry,” in Food Industry. Editors Benjamin V. (Brazil: Scientific, Health and Social Aspects of the Food Industry), 83–94. 10.5772/31256

[B151] Su Z. Lu F. P. Gao Q. Liu X. G. Wang B. Z. Niu T. (2010). “Cloning and expression of a thermostable pullulanase gene from *Thermotogamaritima* MSB8 in Bacillus subtilis WB600,” in Proceedings of the 2010 4th International Conference on Bioinformatics and Biomedical Engineering, Chengdu, 2010 Jun 18–20 (IEEE), 1–4.

[B152] Suganthi R. Benazir J. F. Santhi R. Ramesh Kumar V. Hari A. Meenakshi N. (2011). Amylase production by *Aspergillus niger* under solid state fermentation using agroindustrial wastes. Int. J. Eng. Sci. Technol. 3 (2), 1756–1763.

[B153] Sunna A. Moracci M. Rossi M. Antranikian G. (1997). Glycosyl hydrolases from hyperthermophiles. Extremophiles 1 (1), 2–13. 10.1007/s007920050009 9680331

[B155] Swinkels J. J. M. (1985). Composition and properties of commercial native starches. Starch‐Stärke 37 (1), 1–5. 10.1002/star.19850370102

[B156] Taniguchi H. Honnda Y. (2009). Encyclopedia of microbiology. Oxford: Academic Press, 159–173.

[B157] Tiruneh A. T. Negatu A. A. Satheesh N. (2021). Effect of anchote (*Coccinia abyssinica*) and potato starch addition on colloidal stability of pineapple juice. Int. J. Food Sci. 2021, 1–12. 10.1155/2021/6615273 PMC8112957 34055968

[B158] Toor K. J. Ahmad N. Muhammad M. A. Rashid N. (2020). TK-PUL, a pullulan hydrolase type III from *Thermococcus kodakarensis*, a potential candidate for simultaneous liquefaction and saccharification of starch. Amylase 4 (1), 45–55. 10.1515/amylase-2020-0004

[B159] Torres‐Salas P. del Monte‐Martinez A. Cutiño‐Avila B. Rodriguez‐Colinas B. Alcalde M. Ballesteros A. O. (2011). Immobilized biocatalysts: Novel approaches and tools for binding enzymes to supports. Adv. Mat. 23 (44), 5275–5282. 10.1002/adma.201101821 22299142

[B160] Tufvesson P. Törnvall U. Carvalho J. Karlsson A. J. Hatti-Kaul R. (2011). Towards a cost-effective immobilized lipase for the synthesis of specialty chemicals. J. Mol. Catal. B Enzym. 68 (2), 200–205. 10.1016/j.molcatb.2010.11.004

[B161] Turkenburg J. P. Brzozowski A. M. Svendsen A. Borchert T. V. Davies G. J. Wilson K. S. (2009). Structure of a pullulanase from *Bacillus acidopullulyticus* . Proteins Struct. Funct. bioinform. 76, 516–519. 10.1002/prot.22416 19382205

[B162] Upadek H. O. R. S. T. Kottwitz B. E. A. T. R. I. X. (1997). Application of amylases in detergents. Surfactant Sci. Ser. 212, 203.

[B163] Van Der Maarel M. J. Van der Veen B. Uitdehaag J. C. Leemhuis H. Dijkhuizen L. (2002). Properties and applications of starch-converting enzymes of the α-amylase family. J. Biotechnol. 94 (2), 137–155. 10.1016/s0168-1656(01)00407-2 11796168

[B164] Varzakas T. Labropoulos A. Anestis S. (Editors) (2012). Sweeteners: Nutritional aspects, applications, and production technology (New York: CRC Press).

[B165] Velhal C. Sant M. Das S. Kulkarni C. (2014). Production of pullulanase using novel organic substrates. Int. J. Sci. Technol. Res. 3 (3), 94–97.

[B166] Verhue W. Hers H. G. (1966). A study of the reaction catalysed by the liver branching enzyme. Biochem. J. 99 (1), 222–227. 10.1042/bj0990222 4290551 PMC1264978

[B167] Verma V. (2019). Enzymes Market to overtake 400 kilo tons by 2024. Available at: https://www.gminsights.com/industry-analysis/enzymes-market .

[B168] Wackett L. P. (2011). Microbial commercial enzymes: An annotated selection of World Wide Web sites. Microb. Biotechnol. 4, 548–549. 10.1111/j.1751-7915.2011.00274.x PMC6681399 31380613

[B169] Wallenfels K. Bender H. Rached J. R. (1966). Pullulanase from *Aerobacter aerogenes;* production in a cell-bound state. Purification and properties of the enzyme. Biochem. Biophys. Res. Commun. 22 (3), 254–261. 10.1016/0006-291x(66)90474-8 5938919

[B170] Wang M. Hu H. Zhang B. Zheng Y. Wu P. Lu Z. (2022). Discovery of a new microbial origin cold-active neopullulanase capable for effective conversion of pullulan to panose. Int. J. Mol. Sci. 23 (13), 6928. 10.3390/ijms23136928 35805929 PMC9267027

[B171] Wangpaiboon K. Charoenwongpaiboon T. Klaewkla M. Field R. A. Panpetch P. (2023). Cassava pullulanase and its synergistic debranching action with isoamylase 3 in starch catabolism. Front. Plant Sci. 14, 1114215. 10.3389/fpls.2023.1114215 36778707 PMC9911869

[B172] Wei W. Ma J. Chen S. Q. Cai X. H. Wei D. Z. (2015). A novel cold-adapted type I pullulanase of *paenibacilluspolymyxa* nws-pp2: *In vivo* functional expression and biochemical characterization of glucans hydrolyzates analysis. BMC Biotechnol. 15 (1), 96–13. 10.1186/s12896-015-0215-z 26481143 PMC4615870

[B173] Whelan W. J. (1960). The action patterns of α‐amylases. Starch‐Stärke 12 (12), 358–364. 10.1002/star.19600121202

[B174] Woolston B. M. Jenkins D. J. Hood-Pishchany M. I. Rakoff-Nahoum S. Balskus E. P. (2021). Characterization of vaginal microbial enzymes identifies amylopullulanases that support growth of *Lactobacilluscrispatus* on glycogen. BioRxiv 19, 4. 10.1101/2021.07.19.452977

[B175] Wu Y. Huang S. Liang X. Han P. Liu Y. (2022). Characterization of a novel detergent-resistant type I pullulanase from *Bacillus megaterium* Y103 and its application in laundry detergent. Prep. Biochem. Biotechnol., 1–7. 10.1080/10826068.2022.2134890 36271878

[B176] Xu J. Cui W. Cheng J. J. Stomp A. M. (2011). Production of high-starch duckweed and its conversion to bioethanol. Biosyst. Eng. 110 (2), 67–72. 10.1016/j.biosystemseng.2011.06.007

[B177] Xu J. Ren F. Huang C. H. Zheng Y. Zhen J. Sun H. (2014). Functional and structural studies of pullulanase from *Anoxybacillus* sp. LM18-11. Proteins 82 (9), 1685–1693. 10.1002/prot.24498 24375572

[B178] Xu P. Zhang S. Y. Luo Z. G. Zong M. H. Li X. X. Lou W. Y. (2021). Biotechnology and bioengineering of pullulanase: State of the art and perspectives. World J. Microbiol. Biotechnol. 37, 43. 10.1007/s11274-021-03010-9 33547538

[B179] Yang X. Zhu L. Jiang L. Xu Q. Xu X. Huang H. (2015). Optimization of bioconversion process for trehalose production from enzymatic hydrolysis of kudzu root starch using a visualization method. *Bioresour.* Bioprocess. 2 (1), 37. 10.1186/s40643-015-0065-5

[B180] Yu C. Sun C. Yu L. Zhu M. Xu H. Zhao J. (2014). Comparative analysis of duckweed cultivation with sewage water and SH media for production of fuel ethanol. PloS one 9 (12), e115023. 10.1371/journal.pone.0115023 25517893 PMC4269401

[B181] Zhang H. Jin Z. (2011). Preparation of resistant starch by hydrolysis of maize starch with pullulanase. Carbohydr. Polym. 83 (2), 865–867. 10.1016/j.carbpol.2010.08.066

[B182] Zhang K. Su L. Duan X. Liu L. Wu J. (2017). High-level extracellular protein production in *Bacillus subtilis*using an optimized dual-promoter expression system. Microb. Cell Fact. 16, 32. 10.1186/s12934-017-0649-1 28219382 PMC5319110

[B183] Zhang K. Su L. Wu J. (2022). Enhancing extracellular pullulanase production in *Bacillus subtilis* through dltB disruption and signal peptide optimization. Appl. Biochem. Biotechnol. 194, 1206–1220. 10.1007/s12010-021-03617-6 34652585

[B184] Zhang L. Zhu X. Zheng S. Sun H. (2009). Photochemical preparation of magnetic chitosan beads for immobilization of pullulanase. Biochem. Eng. J. 46 (1), 83–87. 10.1016/j.bej.2009.04.024

[B185] Zhang S. Y. Guo Z. W. Wu X. L. Ou X. Y. Zong M. H. Lou W. Y. (2020a). Recombinant expression and characterization of a novel cold-adapted type I pullulanase for efficient amylopectin hydrolysis. J. Biotechnol. 313, 39–47. 10.1016/j.jbiotec.2020.03.007 32198062

[B186] Zhang Y. Nie Y. Zhou X. Bi J. Xu Y. (2020b). Enhancement of pullulanase production from recombinant *Bacillus subtilis* by optimization of feeding strategy and fermentation conditions. Amb. Express 10, 11. 10.1186/s13568-020-0948-5 31955316 PMC6969872

[B187] Zona R. Chang‐Pi‐Hin F. O'Donohue M. J. Janeček Š. (2004). Bioinformatics of the glycoside hydrolase family 57 and identification of catalytic residues in amylopullulanase from Thermococcus hydrothermalis: Bioinformatics and mutagenesis of GH-57. Thermococcushydrothermalis Eur. J. Obiochem. 271 (14), 2863–2872. 10.1111/j.1432-1033.2004.04144.x 15233783

[B188] Zou C. Duan X. Wu J. (2016a). Efficient extracellular expression of *bacillusderamifcans*pullulanase in *brevibacilluschoshinensis* . J. Ind. Microbiol. Biotechnol. 43, 495–504. 10.1007/s10295-015-1719-1 26707948

[B189] Zou C. Duan X. Wu J. (2014). Enhanced extracellular production of recombinant *Bacillusderamificans*pullulanase in *Escherichia coli* through induction mode optimization and a glycine feeding strategy. BioresourceTechnology 172, 174–179. 10.1016/j.biortech.2014.09.035 25261864

[B190] Zou C. Duan X. Wu J. (2016b). Magnesium ions increase the activity of *Bacillus deramifcans* pullulanase expressed by *Brevibacillus choshinensis* . Appl. Microbiol. Biotechnol. 100, 7115–7123. 10.1007/s00253-016-7386-y 27026175

