# Supplementary material for: Pullulanase: unleashing the power of enzyme with a promising future in the food industry
Source: Front Bioeng Biotechnol. 2023 Jun 28;11:1139611. doi: 10.3389/fbioe.2023.1139611 (PMC10337586; doi:10.3389/fbioe.2023.1139611)
Supplement: Supplementary file 1 [file DataSheet1.docx]

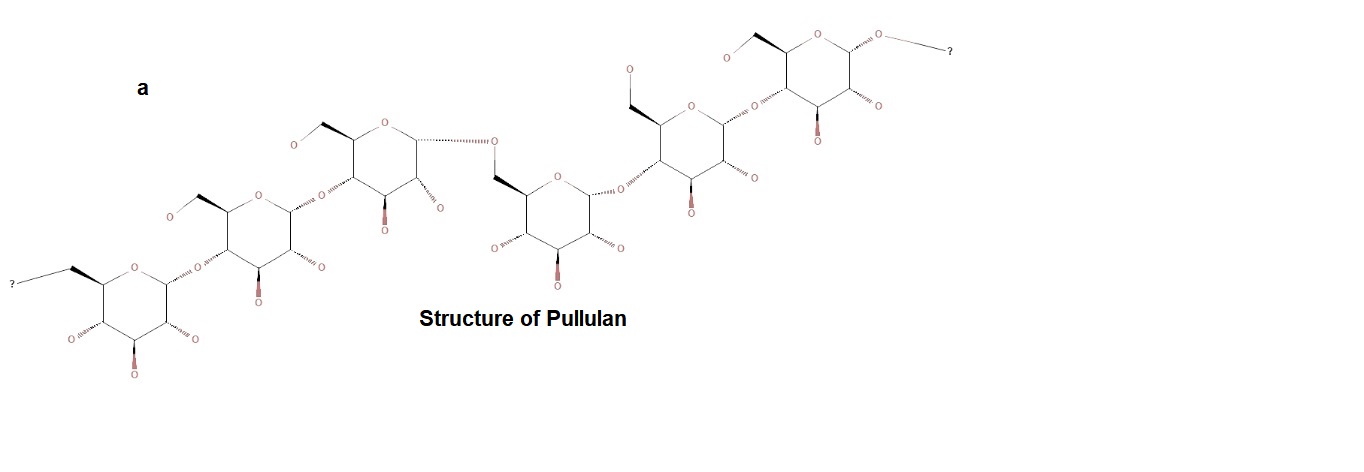


**Fig. S1 A-Structure of Pullulan**


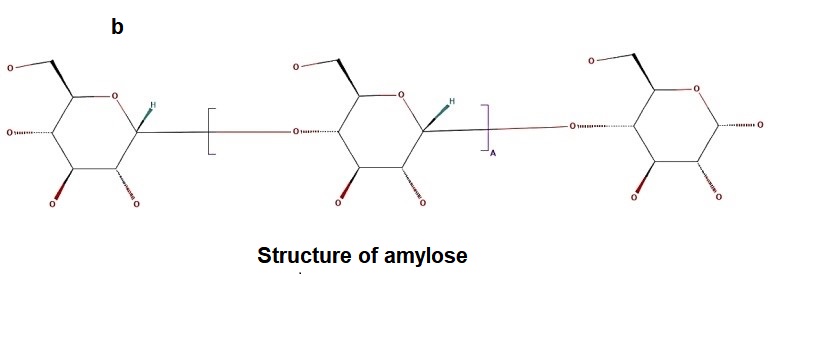


**Fig. S1 -B- Structure of amylose**


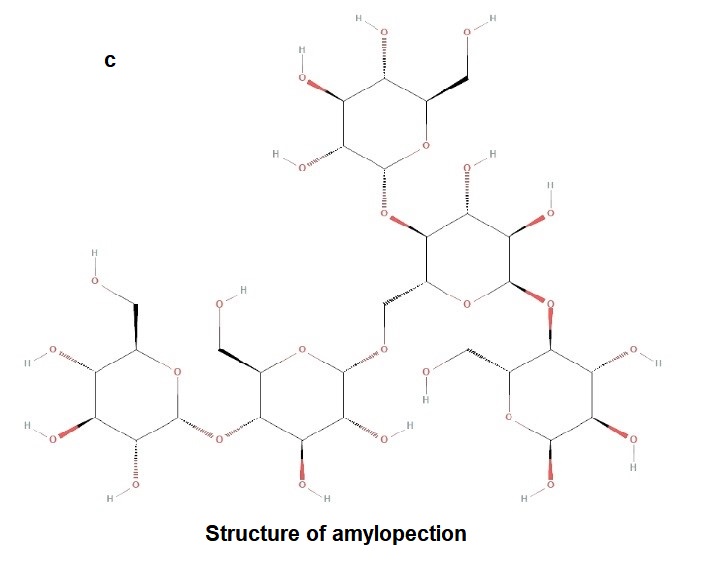


**Fig. S1 C-Structure of amylopectin**


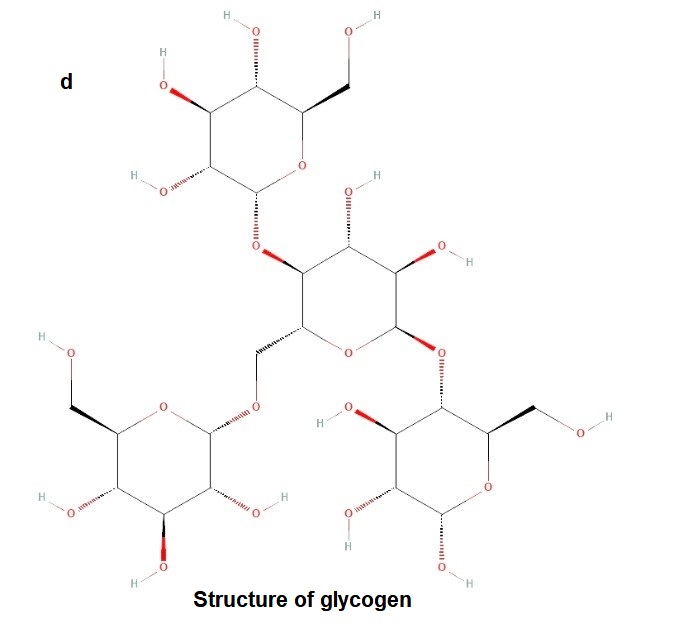


**Fig. S1 D- Structure of glycogen**
